# External Validation of e‐ASPECTS Software for Interpreting Brain CT in Stroke

**DOI:** 10.1002/ana.26495

**Published:** 2022-09-23

**Authors:** Grant Mair, Philip White, Philip M. Bath, Keith W. Muir, Rustam Al‐Shahi Salman, Chloe Martin, David Dye, Francesca M. Chappell, Adam Vacek, Rüdiger von Kummer, Malcolm Macleod, Nikola Sprigg, Joanna M. Wardlaw

**Affiliations:** ^1^ Centre for Clinical Brain Sciences University of Edinburgh Edinburgh UK; ^2^ Translational and Clinical Research Institute Newcastle University and Newcastle upon Tyne Hospitals NHS Trust Newcastle upon Tyne UK; ^3^ Stroke Trials Unit, Mental Health & Clinical Neuroscience University of Nottingham Nottingham UK; ^4^ School of Psychology & Neuroscience University of Glasgow Glasgow UK; ^5^ Department of Neuroradiology University Hospital, Technische Universität Dresden Dresden Germany; ^6^ UK Dementia Research Institute Centre at the University of Edinburgh Edinburgh UK

## Abstract

**Objective:**

The purpose of this study was to test e‐ASPECTS software in patients with stroke. Marketed as a decision‐support tool, e‐ASPECTS may detect features of ischemia or hemorrhage on computed tomography (CT) imaging and quantify ischemic extent using Alberta Stroke Program Early CT Score (ASPECTS).

**Methods:**

Using CT from 9 stroke studies, we compared software with masked experts. As per indications for software use, we assessed e‐ASPECTS results for patients with/without middle cerebral artery (MCA) ischemia but no other cause of stroke. In an analysis outside the intended use of the software, we enriched our dataset with non‐MCA ischemia, hemorrhage, and mimics to simulate a representative “front door” hospital population. With final diagnosis as the reference standard, we tested the diagnostic accuracy of e‐ASPECTS for identifying stroke features (ischemia, hyperattenuated arteries, and hemorrhage) in the representative population.

**Results:**

We included 4,100 patients (51% women, median age = 78 years, National Institutes of Health Stroke Scale [NIHSS] = 10, onset to scan = 2.5 hours). Final diagnosis was ischemia (78%), hemorrhage (14%), or mimic (8%). From 3,035 CTs with expert‐rated ASPECTS, most (2084/3035, 69%) e‐ASPECTS results were within one point of experts. In the representative population, the diagnostic accuracy of e‐ASPECTS was 71% (95% confidence interval [CI] = 70–72%) for detecting ischemic features, 85% (83–86%) for hemorrhage. Software identified more false positive ischemia (12% vs 2%) and hemorrhage (14% vs <1%) than experts.

**Interpretation:**

On independent testing, e‐ASPECTS provided moderate agreement with experts and overcalled stroke features. Therefore, future prospective trials testing impacts of artificial intelligence (AI) software on patient care and outcome are required before widespread implementation of stroke decision‐support software. ANN NEUROL 2022;92:943–957

Artificial intelligence (AI) software is increasingly available to assist clinicians interpret medical imaging. In stroke, where rapid image interpretation guides therapy, several products are used clinically. However, radiology AI is evolving rapidly, and standardized evaluation methods are lacking. Systematic reviews of radiology AI software raise concerns about bias, and the need for consistent external validation,^1^, ^2^ including for stroke.^3^, ^4^ One review of all AI software for radiology included 82 studies, 69 provided sufficient data to test accuracy, whereas only 14 compared AI with health care professionals *in the same sample* (none were in stroke).^1^ In another review of software to evaluate brain computed tomography (CT) in ischemic stroke with 68 studies, 38 reported insufficient data on stroke, patient demographics, or clinical testing.^3^

Within hours of stroke onset, when treatment is most effective, signs of ischemia on CT imaging are often subtle yet lesion extent may guide treatment decisions. The Alberta Stroke Program Early CT Score (ASPECTS) aids visual assessment by quantifying the extent of middle cerebral artery (MCA) territory ischemic injury (CT hypoattenuation and/or swelling) in 10 regions (a score of 10 is normal, and 0 means the entire territory is affected).^5^ Brainomix Ltd. (Oxford, UK) developed AI software (e‐ASPECTS) to automatically identify CT features of stroke, including (1) ASPECTS, (2) hyperattenuated MCA (indicating arterial thrombus), and (3) intracranial hemorrhage (ICH).

Following a PubMed search (to August 6, 2021) using the company and software names, and review of evidence published on the company website, we identified 24 studies in English (excluding abstracts) evaluating e‐ASPECTS, Supplementary Table S1. The median number of patients in these studies was 125, and over half (14/24) declared financial conflicts of interest with Brainomix. There was no prospective randomized testing. Twenty studies included patients with proven ischemic stroke only, thus precluding the assessment of true negative cases. Most of the studies excluded poor‐quality CT (14 excluded, and 7 did not specify), 17 of 24 did not report software failures, and only 4 of 24 tested the impact of patient or imaging factors on software performance.

We established the “Real‐World Independent Testing of e‐ASPECTS Software” (RITeS) study to provide a large scale, clinically representative, and objective assessment of e‐ASPECTS for identifying relevant features on CT brain imaging in patients with stroke.

## Methods

### Study Design

We used data from 9 completed clinical trials or observational studies of patients with stroke in which CT had been assessed by panels of masked experts and a final diagnosis of stroke type determined.^6^, ^7^, ^8^, ^9^, ^10^, ^11^, ^12^, ^13^, ^14^

In a secondary analysis of these prospectively collected data, we processed the CTs using e‐ASPECTS to compare the expert scan assessments and final diagnoses with e‐ASPECTS results for the detection of acute ischemic features or ICH.

Following development of our research plan, we signed a software licensing agreement with Brainomix for use of e‐ASPECTS and paid for the software using academic funds. We agreed to separate testing into 2 types: (1) where software is used on the intended population, and (2) other clinical scenarios where software *might* be used. We thus used 2 overlapping populations:

1. “Target population”: Patients with possible ischemic stroke but no alternative pathology on CT (ie, potential candidates for thrombolysis). Here, we included patients with a final diagnosis of ischemic stroke or stroke mimic without a CT‐identifiable cause, and compared ASPECTS provided by experts versus software.
2. “Representative population”: To simulate hospital‐presenting patients with suspected stroke, we enriched our dataset to include realistic proportions of patients with a final diagnosis of ischemic stroke, ICH, and stroke mimics, and tested the diagnostic accuracy of software versus experts for identifying CT features that might account for stroke symptoms.

We report our results according to Transparent Reporting of a multivariable prediction model for Individual Prognosis Or Diagnosis (TRIPOD),^15^ but due to overlap of our research methods, we also consider other reporting standards, see Appendices S1–S3.

### Patient Population

We analyzed CT brain scans performed soon after stroke onset from 7 national or international multicenter randomized controlled trials (RCTs) and 2 single‐center prospective observational studies. These 9 studies recruited patients with acute stroke since May 2000: and one is ongoing.^16^ Six included ischemic stroke,^6^, ^7^, ^8^, ^9^, ^10^, ^11^ 2 were ICH,^12^, ^14^ and one included all stroke or stroke mimics.^13^ Of the RCTs, 2 tested thrombolytics,^6^, ^8^ one tested imaging strategies,^11^ one tested thrombectomy,^9^ one tested hypothermia,^7^ one tested blood pressure lowering,^13^ and one tested antithrombotic drugs after ICH^12^; of the observational studies, one studied hemorrhagic,^14^ and the other studied ischemic stroke.^10^ We were unable to secure approval in time to include a tenth study as initially proposed.^16^, ^17^

All 9 included studies had research ethical approval and obtained informed consent for all participants.

### Clinical Data Assessment

All 9 studies centrally recorded patient demographics, stroke severity, time elapsed from stroke onset to CT, allocated treatment in the RCTs, and functional outcome.

Final diagnosis (ischemic stroke, ICH, and stroke mimic) was determined similarly in each study by central expert event adjudication, which included the local principal investigator's diagnosis, central masked expert panel review of baseline and follow‐up imaging, and all other study data.

### Sample for RITeS

We estimated that 725 patients were needed to determine whether e‐ASPECTS is noninferior to experts (5% noninferiority limit).^16^ To improve the precision of diagnostic accuracy estimates and power for subgroup analyses, we increased our sample size by including all baseline CTs available to RITeS. We did not otherwise select patients for inclusion; we did not exclude patients with low‐quality imaging, with ischemic lesions outside the MCA territory, or if final diagnosis was stroke mimic.

To assess whether our sample was clinically representative of patients admitted to the hospital with stroke, we prespecified that age, sex, stroke severity, and time since symptom onset in RITeS would be similar to the UK Sentinel Stroke National Audit Programme (SSNAP; April 2018–March 2019, www.strokeaudit.org), pooled RCT, and registry data.^16^

For “target population” testing, we excluded patients with hemorrhage or stroke mimic caused by a structural lesion. For “representative population” testing, we included all patients in RITeS.

### Expert Image Assessment

Prior to RITeS, the CTs in the original 9 studies had been rated by central expert panels (total 24 experts with crossover among studies, one expert report per scan), masked to follow‐up imaging and most other clinical data. Two studies provided experts with the side affected by stroke,^10^, ^13^ and one provided stroke onset time.^13^ In 2 studies, experts reviewed CT and concurrent angiography together.^9^, ^11^ In the study with the largest contribution to RITeS, experts reviewing CT were masked to all other data.^8^ For 6 studies,^7^, ^8^, ^9^, ^11^, ^12^, ^13^ 20 experts performed imaging assessment using the same validated online viewing platform (SIRS 1/2, https://sirs2.ccbs.ed.ac.uk/sirs2).^18^ CT was scored for: ASPECTS^5^; ischemic injuries in all arterial territories (based on visible hypoattenuation and/or swelling of brain); presence of hyperattenuated arteries; ICH location and size; structural mimics; and pre‐stroke brain changes (atrophy, leukoaraiosis, and old stroke lesions).^18^ CT image quality was recorded as good, moderate, or poor. We have previously tested inter‐rater agreement for 7 experts using SIRS: Krippendorff's Alpha (k‐alpha) was 0.66 for identifying ischemia, and 0.56 for ASPECTS.^19^ Two other ischemic stroke studies in RITeS assessed CT for ischemic brain lesions, ASPECTS, and hyperattenuated arteries only.^6^, ^10^ Two RITeS studies evaluating hemorrhagic stroke included assessment of hemorrhage location and size but not ASPECTS.^12^, ^14^

### Image Software Processing

We processed batches of 10 CT scans using the Digital Imaging and Communications in Medicine (DICOM) format on the cloud‐based Brainomix platform (https://brainomix.com, versions 9–10). We selected the earliest scan after stroke for each patient and, to be as close as possible to software specifications, used the thinnest slice axial plane CT constructed for soft tissue viewing.

We recorded all upload and processing outcomes. Where a scan did not process, we made further attempts (with alternative DICOM image sets where available). Processing was considered “successful” when software provided an ASPECTS result or when arterial hyperattenuation or hemorrhage were detected. The e‐ASPECTS allows users to input the side affected by stroke. We manually included this information for a subset of the target population (35%, 1,052 of 3,035) where side information was available and compared before and after results. We exported e‐ASPECTS results to spreadsheets for analysis. We did not review the e‐ASPECTS imaging overlays for every case but inspected batch outputs during processing. We also reviewed imaging overlays when uploading affected side data, and in cases that did not process normally.

Once CT processing was complete, we randomly selected a subsample of 100 scans for repeatability testing, stratified by study that had been successfully processed by e‐ASPECTS. To ensure e‐ASPECTS did not recognize recurrent DICOM meta‐data at repeat testing, we created new unique scan identifiers for this subsample with modiCAS DICOM anonymizer (Erlangen, Germany).

### Primary Outcomes

1. ASPECTS score agreement between experts and e‐ASPECTS (including the side affected) in the target population.
2. Diagnostic accuracy of experts and e‐ASPECTS for identifying CT features that might account for stroke symptoms (ie, signs of ischemia or hemorrhage) in the representative population, which is outside the intended use of the software.

### Secondary Outcomes

1. Proportion of scans successfully processed by e‐ASPECTS; factors associated with processing success and accuracy.
2. Repeatability of e‐ASPECTS results on the subset of scans presented twice.

### Testing and Statistics

We have published the RITeS Statistical Analysis Plan, summarized here.^16^ We followed an “intention‐to‐process” methodology regardless of whether the scan processing was successful.

We principally used diagnostic accuracy statistics to compare e‐ASPECTS and expert results. Reference standards varied by test. To assess e‐ASPECTS for identifying acute MCA territory ischemic injury at clinically relevant thresholds (ASPECTS 10 vs 0–9; 8–10 vs 0–7; and 6–10 vs 0–5), we used masked expert ASPECTS at baseline as the reference. To compare e‐ASPECTS versus masked experts at baseline for identifying features of ischemia (ischemic brain injury or hyperattenuating arteries or both) or ICH as the cause of stroke, we used the final diagnosis as the reference. To account for individual study result clustering and to assess variation within/between contributing studies, we included random‐effects bivariate meta‐analysis modeling estimates of sensitivity and specificity. We used the Prediction model Risk Of Bias ASsessment Tool (PROBAST) to assess the risk of bias and applicability of our testing.^20^ To aid understanding, we summarized results as proportions with or without expert agreement per 100 patients.

We compared expert and software ASPECTS using Bland–Altman plots and prespecified that scores would be considered “equivalent” if within 2 points and for the same cerebral hemisphere.^16^ We assessed expert‐software agreement with k‐alpha. Both methods assess agreement while controlling for inherent result correlation. To compare with previous work, we used Matthews Correlation Coefficient (MCC) and assessed noninferiority.^21^ We prespecified that e‐ASPECTS would be noninferior if the 90% confidence interval (CI) lower limit for the difference (e‐ASPECTS minus expert results) was greater than −5%.^16^ For assessing factors associated with expert‐software agreement of ASPECTS and the diagnostic accuracy of software to detect MCA ischemia (compared to experts), we prespecified test variables and their subgroups.^16^ We checked for collinearity in multivariable testing (variance inflation factors >5). We did not impute, but report missing data.

We conducted sensitivity analyses of our primary outcomes for randomly selected subgroups:

1. Of the target population, with
  - Balanced representation from all RITeS studies by excluding excess cases from studies with more than double the median case number per trial, and
  - Hyperattenuating internal carotid or middle cerebral arteries as a surrogate for large vessel occlusion (ie, not randomly selected).^22^
2. Of the representative population, where stroke mimics without structural lesions represent 26% of the total (as identified in RIGHT‐2^13^).

For repeatability testing, we assessed the number of matched initial versus repeat e‐ASPECTS results.

We used SPSS, IBM Corporation (Armonk, NY, USA) for most analyses except meta‐analysis,^23^ where we used MetaDTA (version 2.0: https://crsu.shinyapps.io/dta_ma/) and Review Manager (RevMan 5.4, The Cochrane Collaboration).

## Results

### Study Sample Characteristics

**FIGURE 1:**
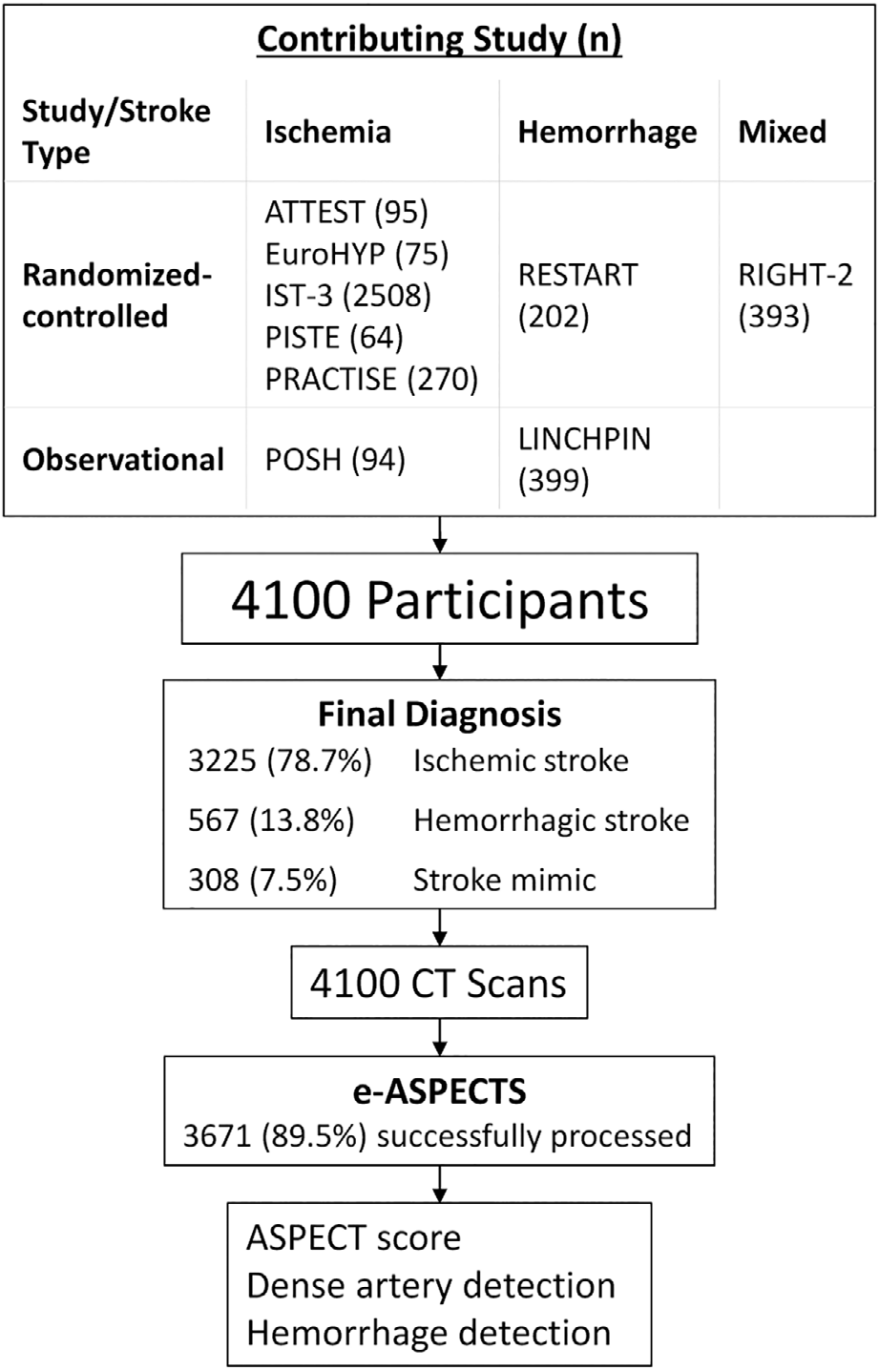
Flowchart of contributing trials’ participants, final diagnosis, brain imaging, and image processing in RITeS. ASPECTS = Alberta Stroke Program Early CT Score; CT = computed tomography.

Of 5,967 participants in 9 studies, RITeS included 4,100, recruited and scanned between June 2003 and May 2018 (see Fig 1). A total of 1,867 were not available to RITeS, 3 studies shared only a subset of cases (994/2,281 from LINCHPIN, RESTART, and RIGHT‐2), whereas CT was not available centrally for some cases (580). Of 4,100 available to RITeS: 2,069 (51%) were women, median age was 78 years (interquartile range [IQR] = 68–85 years), median NIHSS 10 (IQR = 6–16); median time elapsed since stroke onset 2.5 hours (IQR = 1.8–3.8); and final diagnosis was ischemic stroke (3,225, 78%), ICH (567, 14%), or mimic (308, 8%). From eligible patients, 1,701 of 3,218 (53%) were treated with intravenous thrombolytic and 35 of 79 (44%) with thrombectomy (only available in 2/9 studies). RITeS population characteristics were within the ranges presented by prespecified comparison datasets,^16^ indicating our sample is clinically representative without modification. Risk of bias was low (PROBAST) with no applicability concerns, Appendix S4.

From 4,100 CTs included in RITeS, experts identified 1,214 (30%) acute ischemic brain lesions, most were classed as “subtle” (873/1,214, 72%); and, where assessed, identified arterial hyperattenuation in 768 of 3,443 (22%). Combining both features, most ischemic strokes affected the MCA territory (1,390/1,517, 92%). Most hemorrhages were intracerebral (567/643, 88%). Eighty‐seven patients (2%) had alternative structural causes for stroke symptoms (221/308 mimics did not), whereas 4% (167) had incidental findings not related to stroke. Where assessed, most scans had at least one case of leukoaraiosis, atrophy or old stroke lesions (3,402/3,912, 87%), and were judged to be “good to moderate” quality (3,708/3,917, 95%) with image slice thickness ≤5 mm (3,345/3,986, 84%; Table 1).

**TABLE 1:** Radiological Characteristics for all 4,100 Participant Scans in RITeS

Radiological Feature	N (%)
Ischemic brain changes	
Hypoattenuation	
None	2,886 (70.4%)
Subtle (loss of grey‐white margins)	873 (21.3%)
Obvious (darker than normal white matter)	210 (5.1%)
Present but no visibility score	131 (3.2%)
Swelling^a^ (n = 1,148)	
None	486 (42.3%)
Sulcal effacement only	517 (45.0%)
Sulcal and ventricular effacement	144 (12.5%)
Sulcal, ventricular, and basal cistern effacement	1 (<0.1%)
Hyperattenuating arteries^a^ (n = 3,443)	
None	2,675 (77.7%)
ICA/MCA	730 (21.2%)
Other intracranial artery	38 (1.1%)
Ischemia location	
None	2,595 (63.2%)
MCA territory	1,390 (33.8%)
Other cerebral	54 (1.3%)
Brainstem or cerebellum	45 (1.1%)
Multiple locations	25 (0.6%)
Hemorrhage location	
None	3,457 (84.3%)
Deep	323 (7.9%)
Lobar	312 (7.6%)
Intraventricular	255 (6.2%)
Extra‐axial	274 (6.7%)
Hemorrhage cause (n = 643)	
Cerebral small vessel disease	567 (88.2%)
Vascular lesion (malformation and aneurysm)	44 (6.8%)
Underlying infarct	5 (0.8%)
Trauma	6 (0.9%)
Other underlying lesion	21 (3.3%)
Structural stroke mimic	
None	4,022 (97.9%)
Tumor	40 (1.0%)
Aneurysm	26 (0.6%)
Arteriovenous malformation	12 (0.3%)
Cavernous malformation	6 (0.1%)
Collection	2 (<0.1%)
Other	1 (<0.1%)
Incidental findings—Not related to stroke symptoms^a^ (n = 3,921)	
None	3,753 (95.7%)
Developmental/ acquired	91 (2.3%)
Previous surgery/ old trauma	27 (0.7%)
Tumor	26 (0.7%)
Hygroma	12 (0.3%)
Other	11 (0.3%)
Pre‐stroke brain changes^a^ (n = 3,912)	
None	510 (13.0%)
Atrophy	3,188 (81.5%)
Leukoaraiosis	2,152 (55.0%)
Old stroke lesions	1918 (49.0%)
Image quality^a^ (n =3,917)	
Good	2,579 (65.8%)
Moderate	1,129 (28.8%)
Poor	209 (5.3%)
CT slice thickness^a^ (n = 3,986)	
Thin (≤1 mm)	1,511 (37.9%)
Medium (>1 mm to ≤5 mm)	1834 (46.0%)
Thick (>5 mm)	641 (16.1%)

^a^ Data not available for all patients, total N presented.

CT = computed tomography; ICA = internal carotid artery; MCA = middle cerebral artery; RITeS = Real‐World Independent Testing of e‐ASPECTS Software.

### Image Processing

Of 4,100 CT uploaded to e‐ASPECTS, 3,671 (90%) were successfully processed. Reasons for incomplete processing of 429 scans were: upload failure (176), cancellation of processing (121), segmentation failure (42), bilateral ischemic changes identified (37), processed as CT angiography (25), unable to read input files (20), internal error (5), and scoring failure (3).

### Primary Outcomes

#### “Target Population” – ASPECTS Score Agreement

**FIGURE 2:**
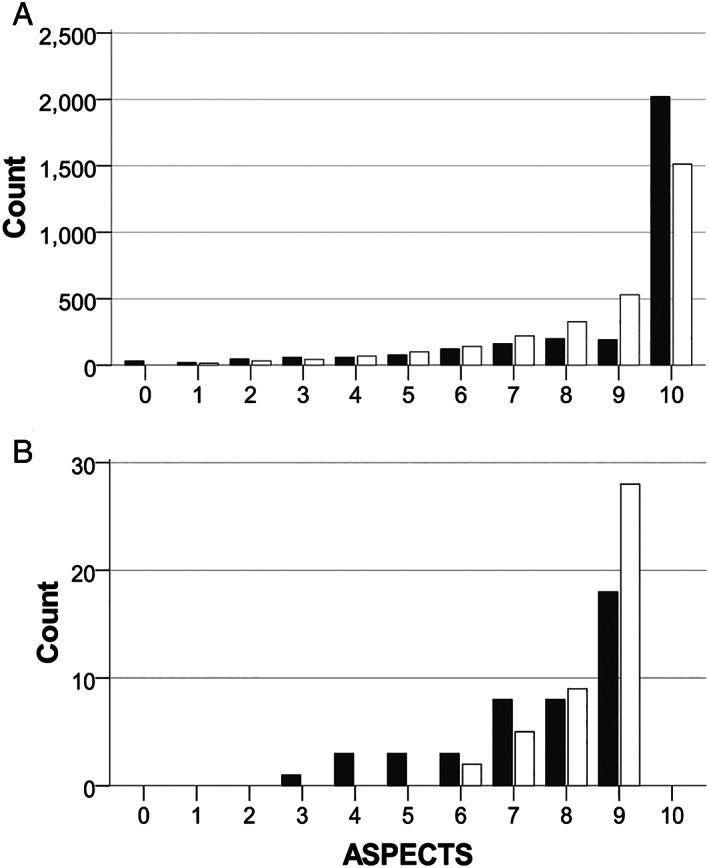
Overlapping histograms comparing ASPECTS results provided by experts versus e‐ASPECTS. Includes 3,035 scans in the “target population” scored by both e‐ASPECTS (open bars) and experts (closed bars). Median scores were 10 (IQR = 8–10) for both groups but score distribution was different on Mann–Whitney *U* testing, *p* < 0.001. (A) Includes 2,991 scans where any detected abnormality (ASPECTS <10) was in the same hemisphere. (B) Includes 44 scans where e‐ASPECTS and experts scored the opposing hemisphere abnormal. ASPECTS = Alberta Stroke Program Early CT Score; IQR = interquartile range.

Figure 2 compares ASPECTS provided by experts versus e‐ASPECTS in patients without hemorrhage or a structural mimic, n = 3,035. Experts were more likely than e‐ASPECTS to report scans as normal (ASPECTS = 10), *p* < 0.001. In pairwise testing, experts and e‐ASPECTS provided the same ASPECTS in 1,406 (46%), whereas cumulatively 2,084 (69%) matched or differed by 1 point and 2,486 (82%) by 2 points. The remaining 505 (17%) differed by >2 points or scored opposing hemispheres (44, 1%). On sensitivity testing in a sample (n = 1,173) where all 9 RITeS studies were numerically balanced, paired scores were better, being identical, ±1, ±2, and >2 ASPECTS points or in opposing cerebral hemispheres in 54%, 75%, 85%, 14%, and 1%, respectively.

In the sample where e‐ASPECTS was given information on the side affected by stroke (n = 1,052), software was less likely to score the opposite hemisphere from experts with (<1%, 3/1,052) versus without (4%, 38/1,052) this knowledge, *p* < 0.0001.

**FIGURE 3:**
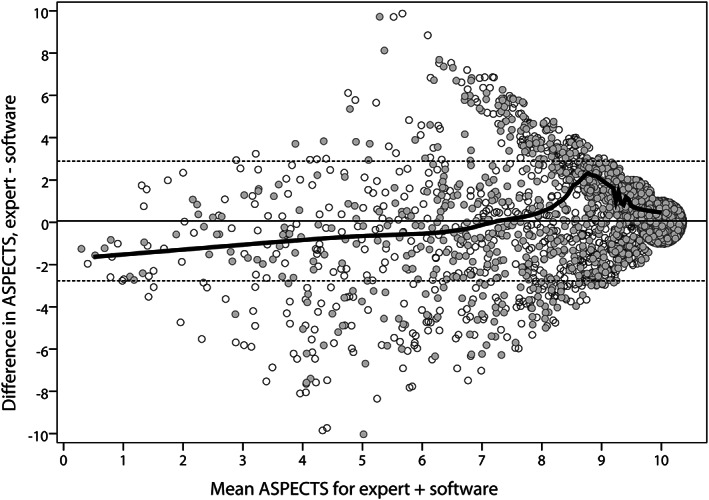
Bland–Altman plot comparing ASPECT scores for all CT brain scans in the “target population” scored by both e‐ASPECTS and experts, n = 3,035. Open and closed circles are from the left and right cerebral hemispheres, respectively. To aid visual representation of data, SPSS “jitter” function applied to minimally displace results that match on both the *x* and *y* axes. Includes locally estimated scatterplot smoothing (LOESS) line of best fit (not affected by jitter). Mean difference in ASPECTS 0.06 points (solid line), SD = 1.44 ASPECTS points (dotted lines ±1.96 SD). ASPECTS = Alberta Stroke Program Early CT Score; CT = computed tomography.

On noninferiority testing, the mean percentage difference between scores (90% CI) was −0.9% (−2.0 to –0.2%), indicating that e‐ASPECTS results were noninferior to experts. Experts and e‐ASPECTS had moderate agreement for left (k‐alpha 0.50, 95% CI = 0.46–0.53) and right (0.49, 95% CI = 0.44–0.54) cerebral ASPECTS. Compared with experts, e‐ASPECTS tended to score larger lesions as smaller, and score smaller lesions as larger (see Fig 3).

The diagnostic accuracy of e‐ASPECTS for identifying acute MCA territory ischemia using ASPECTS thresholds ranged from 66% (ASPECTS 10/0–9) to 90% (ASPECTS 6–10/0–5). As the ASPECTS threshold decreased (larger lesions), e‐ASPECTS sensitivity decreased (74–42%) whereas specificity increased (62–95%). However, the positive and negative predictive values for detecting MCA ischemia were more stable across the thresholds, ~50% and ~90%, respectively, Table 2. On sensitivity testing, only including patients with large vessel occlusion demonstrated as hyperattenuating internal carotid or middle cerebral arteries (n = 683), at the threshold for thrombectomy (ASPECTS 6–10/0–5), e‐ASPECTS was 42% sensitive (95% CI = 35–49%) and 90% specific (95% CI = 87–93%). Summary receiver operating characteristic (ROC) curves suggest ASPECTS 10/0–9 is the most effective binary e‐ASPECTS discriminator compared to experts. The MCC between expert and e‐ASPECTS results by threshold was 0.34 (ASPECTS 10/0–9), 0.41 (ASPECTS 8–10/0–7), and 0.39 (ASPECTS 6–10/0–5).

**TABLE 2:** Diagnostic Accuracy Testing

Test		Comparator	n	TP	TN	FP	FN	Sens	Spec	PPV	NPV	Accuracy
Abnormal e‐ASPECTS^a^	10 versus 0–9	Masked expert at baseline	3,035	753	1,255	768	259	74 (72–77)	62 (60–64)	50 (48–51)	83 (81–84)	66 (64–68)
	8–10 versus 0–7			323	2,136	304	272	54 (50–58)	88 (86–89)	52 (48–55)	89 (88–90)	81 (80–82)
	6–10 versus 0–5			125	2,601	134	175	42 (36–47)	95 (94–96)	48 (43–54)	94 (93–94)	90 (89–91)
Cause of stroke symptoms, CT (e‐ASPECTS)^b^	Detecting ischemic signs^c^	Expert opinion at follow‐up using all data	3,708^d^	1,382	1,252	438	636	68 (66–70)	74 (72–76)	76 (74–77)	66 (65–68)	71 (70–72)
	Detecting hemorrhage			588	2,550	531	39	94 (92–95)	83 (81–84)	53 (51–54)	98 (98–99)	85 (83–86)
Cause of stroke symptoms, CT (masked expert at baseline)	Detecting ischemic signs^c^		4,100	1,318	1751	89	942	58 (56–60)	95 (94–96)	94 (92–95)	65 (64–66)	75 (74–76)
	Detecting hemorrhage			642	3,457	1	0	100 (99–100)	100 (100–100)	100 (99–100)	100 (100–100)	100 (100–100)

Sensitivity (Sens), specificity (Spec), positive predictive value (PPV), negative predictive value (NPV), and accuracy results are provided as % (95% confidence interval).

^a^ “Target population”: For threshold testing, a negative result always included the group with ASPECTS = 10.

^b^ “Representative population” includes cases with imaging features outside software scope: non‐MCA ischemia (116/3,708, 3.1%) and structural stroke mimics (80/3,708, 2.2%).

^c^ Includes ischemic brain lesions or hyperattenuating arteries.

^d^ Includes 37 scans where e‐ASPECTS detected bilateral ischemic lesions.

ASPECTS = Alberta Stroke Program Early CT Score; CT = computed tomography; FN = false negative; FP = false positive; MCA = middle cerebral artery; TN = true negative; TP = true positive.

### “Representative Population” – Diagnostic Accuracy of e‐ASPECTS versus Experts for Identifying Cause of Stroke on CT

When identifying features of ischemic stroke on CT (ischemic brain lesions and/or hyperattenuating arteries), e‐ASPECTS was more sensitive (68% vs 58%) but less specific (74% vs 95%) than experts. Experts had a better positive predictive value (94% vs 76%) and greater accuracy than e‐ASPECTS (75% vs 71%), driven by fewer false positives: experts 2% (89/4100) versus e‐ASPECTS 12% (438/3708), *p* < 0.0001; see Table 2.

For hemorrhage detection, e‐ASPECTS had lower specificity, positive predictive value, and accuracy compared with experts, primarily due to false positive results: experts (1/4,100, <1%), e‐ASPECTS (531/3,708, 14%, 507 with a final diagnosis of ischemia, and 24 with a non‐hemorrhagic stroke mimic), *p* < 0.0001. There were fewer software false negative results for hemorrhage detection (39/3,708, 1%).

Negative predictive values for ischemia (~ 65%) and hemorrhage (~ 100%) detection were very similar between experts and e‐ASPECTS.

On sensitivity testing in a subset where 26% (221/849) had a final diagnosis of stroke mimic without a corresponding structural abnormality on CT (thus within software scope), we include 63% (538/849) with a final diagnosis of ischemia and 11% (90/849) with hemorrhage. In this subset, diagnostic accuracy results for e‐ASPECTS were almost unchanged: for detection of ischemic signs, software sensitivity was 61%, and specificity was 75%; for detection of hemorrhage, software sensitivity was 97%, and specificity was 83%.

**FIGURE 4:**
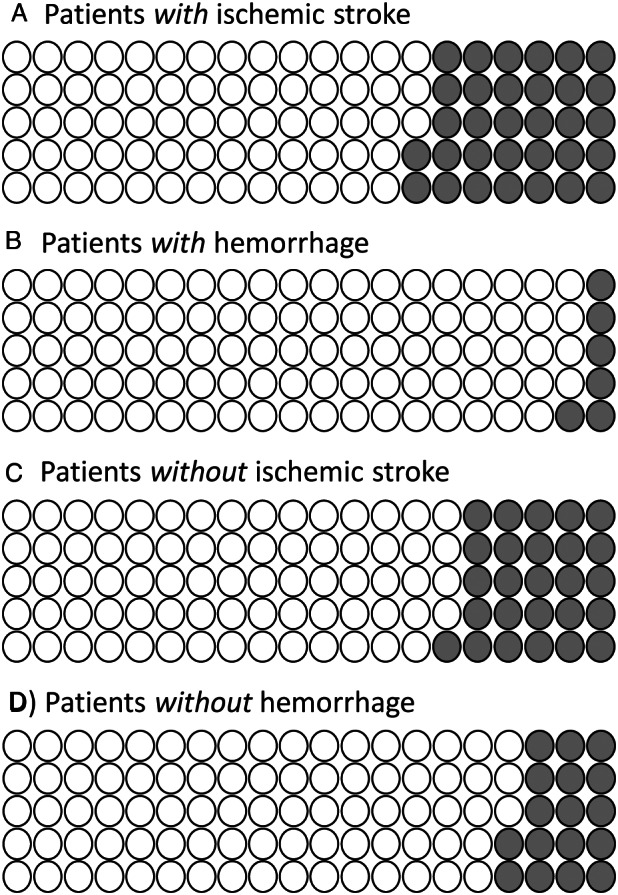
Normalized frequency plots per 100 patients scanned with e‐ASPECTS for identification of any ischemic feature or hemorrhage. These analyses use the “representative population” and include cases with imaging features outside the software manufacturer's recommendation; 3% have ischemia outside the MCA territory, whereas 2% have structural mimics. (A, B), open circles represent true positives, closed circles false negatives. (C, D), open circles represent true negatives, closed circles false positive results. ASPECTS = Alberta Stroke Program Early CT Score; MCA = middle cerebral artery.

Figure 4 shows the potential clinical impact per 100 patients assessed using e‐ASPECTS:

- With ischemic stroke, ischemia will be correctly detected in 68 but missed in 32.
- Without ischemic stroke, ischemia will be incorrectly detected in 26.
- With ICH, hemorrhage will be correctly detected in 94 but missed in 6.
- Without ICH, hemorrhage will be incorrectly detected in 17.

We found low variance in diagnostic accuracy for ischemia and hemorrhage detection within and between contributing RITeS studies (Supplementary Figs S1 and S2).

### Secondary Outcomes

#### Factors Influencing CT Processing Success and Accuracy

Successful processing was most likely with slice thickness 1 to 5 mm and when experts scored the scan quality as “good.” CTs that did not process were more often from older patients who presented to the hospital earlier and were less likely to include ICH (see Table 3). All these variables except age remained significantly associated with processing success on multivariable binary logistic regression, including 3,465 patients with complete demographic and CT slice thickness data (data not shown).

**TABLE 3:** Characteristics of all 4,100 Patients and their CT Scans Processed Successfully versus Unsuccessfully by e‐ASPECTS: Univariable Comparisons. Whole Sample

Variable	Successfully processed n = 3,671	Not successfully processed n = 429	Absolute difference	*p*
Age, yr	78 (68–84)	81 (68–86)	3 yr	0.026
Sex, female	1853 (50.5%)	216 (50.3%)	0.2%	0.960
Time from stroke onset, h	2.5 (1.8–3.8)	2.3 (1.7–3.6)	0.2 h	0.010
NIHSS	9 (6–16)	10 (6–17)	1	0.688
Visible acute lesions				
MCA territory ischemia	1,239 (33.8%)	152 (35.4%)	1.6%	0.491
Ischemia elsewhere	114 (3.1%)	10 (2.3%)	0.8%	0.323
Hemorrhage	636 (17.3%)	15 (3.5%)	13.8%	<0.00001
Mimic	76 (2.1%)	11 (2.6%)	0.5%	0.536
Any pre‐stroke brain changes				
Atrophy	2,852 (77.7%)	336 (78.3%)	0.6%	0.648
Leukoaraiosis	1940 (52.8%)	212 (49.4%)	3.4%	0.191
Old stroke lesions	1709 (46.6%)	209 (48.7%)	2.1%	0.352
Year scan performed	2010 (09–12)	2010 (09–14)	0 yr	0.865
CT slice thickness				
≤1 mm	1,365 (37.2%)	146 (34.0%)	3.2%	0.005
>1 mm ≤5 mm	1706 (46.5%)	128 (29.8%)	16.7%	
>5 mm	600 (16.3%)	41 (9.6%)	6.7%	
Image quality^a^				
Good	2,337 (63.7%)	238 (55.5%)	8.2%	0.0004
Moderate	992 (27.0%)	137 (31.9%)	4.9%	
Poor	172 (4.7%)	34 (7.9%)	3.2%	

Results are median (interquartile range = IQR) or n (%) as appropriate.

^a^ Quality as judged by masked expert.

ASPECTS = Alberta Stroke Program Early CT Score; CT = computed tomography; MCA = middle cerebral artery; NIHSS = National Institutes of Health Stroke Scale.

Most prespecified variables were associated with differences in the experts’ ASPECTS versus e‐ASPECTS on multivariable regression, with increasing patient age, NIHSS, or slice thickness being associated with larger score differences. Expert and e‐ASPECTS scores were more similar when scans were performed later after stroke, when e‐ASPECTS knew the affected side, and when MCA lesions were smaller or when ischemic lesions were outside the MCA territory (see Table 4).

**TABLE 4:** Multivariable Ordinal Logistic Regression Testing Factors Associated with Agreement between e‐ASPECTS and Expert Human Scores

Predictor variables	Difference expert ASPECTS—e‐ASPECTS			Odds ratio	95% confidence interval	Variance inflation factor	*P*
	0 (1,130)	1–2 (932)	3–10 (461)				
Age, yr	79 (67–85)	82 (73–86)	82 (75–87)	1.01	1.00–1.02	1.41	0.013
NIHSS	7 (5–13)	12 (7–18)	17 (10–22)	1.06	1.04–1.07	1.33	<0.001
Time from stroke onset, h	2.5 (1.7–3.6)	2.5 (1.7–3.6)	2.3 (1.6–3.1)	0.93	0.87–0.99	1.08	0.017
e‐ASPECTS knowledge of affected side, yr/n	421 (37%)	308 (33%)	143 (31%)	0.71	0.60–0.83	1.09	<0.001
ASPECT score, human rated	10 (10–10)	10 (8–10)	8 (5–10)	0.74	0.72–0.77	1.54	<0.001
Ischemic location							
MCA	187 (16%)	505 (54%)	293 (64%)	1.47	1.26–1.72	1.40	<0.001
Other, none	943 (84%)	427 (46%)	168 (36%)				
Pre‐stroke brain changes (any), y/n	947 (84%)	813 (87%)	396 (86%)	0.96	0.76–1.21	1.22	0.716
CT slice thickness, mm	4.5 (1.4–5.0)	5 (3.0–6.0)	5 (3.0–7.5)	1.52	1.34–1.71	1.04	<0.001
Image quality							
Good	791 (70%)	606 (65%)	280 (61%)	0.90	0.77–1.06	1.04	0.196
Moderate	296 (26%)	270 (29%)	146 (32%)				
Poor	43 (4%)	56 (6%)	35 (7%)				

Dependent variable was absolute difference between expert ASPECTS less e‐ASPECTS (ie, integers 0–10). Subgroups of ASPECTS difference for presentation only. n = 2,523 due to incomplete demographic and CT data for 1,577 cases. Raw data are median (interquartile range) or n (%) as appropriate.

ASPECTS = Alberta Stroke Program Early CT Score; CT = computed tomography; MCA = middle cerebral artery; NIHSS = National Institutes of Health Stroke Scale.

Diagnostic accuracy of e‐ASPECTS for identifying MCA ischemic lesions varied according to prespecified subgroups: age (accuracy 77% ≤60 years vs 64% >60 years); NIHSS (70% for mild stroke, 64–63% for moderate–severe stroke); when e‐ASPECTS knew affected side (71% vs 64% when unknown); and CT slice thickness (≤1 mm 73% vs >1 mm 63%), whereas hours from stroke onset did not modify accuracy results (65% for <3 and ≥3).

### Repeatability Testing

There were no differences in e‐ASPECTS results on repeat processing for 99 CTs, with 100% match. Operator error excluded one scan (nonidentical image set incorrectly uploaded for repeat).

## Discussion

RITeS is a large independent assessment of e‐ASPECTS software for acute stroke CT and includes almost as many patients as all other prior studies combined. We used clinically relevant patients from 9 prospective studies and expert opinions as the reference standard. RITeS and the contributing studies were rigorously conducted to minimize bias. We tested e‐ASPECTS according to the manufacturer's guidance: restricted to patients with symptoms of stroke, with or without CT features of ischemia but with other structural abnormalities excluded. We also enriched the dataset to include representative proportions of patients with non‐MCA ischemia, hemorrhage, or a final diagnosis of stroke mimic because this may be more like patients hospitalized with *suspected* stroke. This latter analysis is outside the manufacturer's indications for software use. We found software performance was modified by patient and imaging variables.

### Detection of Acute Ischemic Injury

ASPECTS provided by software and experts were reasonably well matched; results were identical for ~ 50% and within ±1 ASPECTS point for up to 75%. As previously shown, we found e‐ASPECTS noninferior to experts in this context.^21^ Software was more likely to find abnormalities, but conversely underestimated the size of larger lesions. Differences between experts and e‐ASPECTS are most relevant if thresholds are used to exclude patients from thrombectomy (ASPECTS <6). Compared with other thresholds we tested, the diagnostic accuracy for e‐ASPECTS was greatest at ASPECTS <6 driven by a high specificity (95%). However, the specificity was slightly reduced (90%) in the subgroup of patients with large vessel occlusion. Our findings suggest that, for patients assessed using e‐ASPECTS compared to expert interpretation alone, 4% (134 false positive results from 3,035) might be miscategorized as ASPECTS <6, and potentially denied highly effective therapy. Two previous studies showed similar results for e‐ASPECTS <6 with misclassifications of 1.6 to 3.4% in smaller (n ~ 60) cohorts.^24^, ^25^

We did not use concurrent CT perfusion or diffusion‐weighted magnetic resonance imaging (MRI) to define a “ground truth.”^26^, ^27^, ^28^ Therefore, software may identify subtle ischemic injury not appreciated by experts. Indeed, software may be more sensitive than experts (68% vs 58%) for correctly detecting ischemic stroke features. However, any improvement in sensitivity is tempered by increased software false positive results compared with experts (12% vs 2%) and, consequently, lesser software specificity (74% vs 95%). The diagnostic accuracy of e‐ASPECTS for detecting MCA ischemia was lower in older patients, those with more severe strokes and larger infarcts, all non‐modifiable features encountered in patients eligible for thrombectomy. However, the diagnostic accuracy of software can be improved if CT image slices are thin (≤1 mm) and when e‐ASPECTS is provided with the side affected by stroke; e‐ASPECTS was more likely to score the “wrong” hemisphere when the affected side was unknown. These are simple modifiable factors that users can optimize, assuming a degree of vigilance to avoid side errors.

Six studies (median n = 98) include diagnostic accuracy results for e‐ASPECTS with expert reference standards comparable to RITeS: sensitivity 14 to 83%, specificity 57 to 99%, and accuracy 67 to 87%.^21^, ^28^, ^29^, ^30^, ^31^, ^32^ One study used an ASPECTS threshold as we did,^32^ the others considered ischemic detection per ASPECTS region for a summed score (ie, 10 × n). However, 2 studies using summed scores did not control for interdependency between different ASPECTS regions in the same patient.^29^, ^30^ As an alternative to accuracy, 3 of 6 studies assessed MCC citing benefits for testing datasets with true positive/negative imbalance.^21^, ^31^, ^32^ Our MCC results are similar (0.34–0.48). For testing agreement between software and experts, our k‐alpha results are like other validated reader‐reliability scoring methods used in 6 studies (median n = 153): kappa (0.25–0.84)^25^, ^33^; intraclass correlation coefficient (0.47–0.87).^33^, ^34^, ^35^, ^36^, ^37^ For all these comparisons, our results tend toward the mid‐lower end of published ranges. We hypothesize the broad representation of our dataset (even in our target population) explains this. Four of the 12 studies discussed here excluded details of the time elapsed since stroke onset. Elapsed time is critical because ischemic brain lesion visibility on CT (and therefore ease of detectability) increases with time. For wider context, in a previous systematic review, we explored agreement between human readers and similar AI software from different manufacturers that also automates ASPECTS. In that analysis, we identified comparable results from three studies (total n = 609) assessing only one other software, and the results range is similar for the other software; intraclass correlation coefficient = 0.45–0.53.^3^ Additionally, at least one small analysis (n = 52) directly compared e‐ASPECTS with another similar software and found no significant difference between them.^29^

### Detection of Hemorrhage

For acute hemorrhage detection, e‐ASPECTS tends to over‐ rather than under‐call. Where the volume of apparent hemorrhage was small, e‐ASPECTS commonly identified both ischemic and hemorrhagic features on the same scan (ischemic results are suppressed if ≥4 ml of hemorrhage is detected). Greater software sensitivity to “‘hyperdense volumes which may indicate bleeding” compared with experts (14% by software here) might trigger additional expert radiology review and thus delay or deny (if expert opinion is not available) appropriate thrombolysis delivery, potentially limiting treatment‐related improvements in patient outcomes. False negative hemorrhage detection (1%) could cause significant clinical worsening if patients with hemorrhage are inappropriately thrombolyzed. Most mimic patients in RITeS had no alternative CT lesion, but some did. Under these conditions (which are beyond software indications for use), there was greater false positive detection. Note, however, that these results did not differ on sensitivity testing with structural mimics excluded.

The potential clinical impact of our diagnostic accuracy results is summarized in Figure 4. Although e‐ASPECTS correctly classifies many CTs with and without ischemia (~ 70–75%) or hemorrhage (~ 85–95%), a substantial proportion of scans are misclassified compared to the final diagnosis. In general, software was better at excluding than identifying stroke imaging features correctly (greater negative predictive values), driven by higher false positive rates compared with experts. In most analyses, experts performed better, except for true ischemic feature detection where software correctly identified more. Thus, whereas experts may find e‐ASPECTS useful for detecting subtle ischemia, they should be aware of false positive feature detection in particular, and always independently assess the CT for hemorrhage. Therefore, we recommend that if e‐ASPECTS is to be used, it is only used strictly as approved by US and European authorities, that is to support users who are already competent at interpreting stroke imaging.^38^ Although it remains to be proven whether and how this support is helpful in real‐time clinical practice. We have not tested the accuracy of combined software‐expert opinion and whether this is better than expert opinion alone. The performance of experienced but non‐expert clinicians with and without software is also relevant. A previous analysis comparing a range of 16 readers with and without e‐ASPECTS who reviewed 60 CT scans found the ASPECTS for both expert and non‐expert reader groups were more similar to 24‐hour gold‐standard scores when using the software.^24^ This observation, and particularly its impact on care, requires prospective testing.

### Impact of Patient and Imaging Factors

We found that image quality and CT slice thickness are important for successful software processing. It is unclear why scans with acute hemorrhage were more likely to process than those without. Differences in image acquisition methods for hemorrhagic versus ischemic stroke studies in RITeS may contribute. Nearly all clinical and imaging variables we tested modified the agreement between expert and software ASPECTS, as shown previously for slice thickness and presence of background brain changes.^30^, ^39^ We were surprised that pre‐stroke brain changes visible on CT (atrophy, leukoaraiosis, and old stroke lesions) were not similarly negatively associated with expert‐software agreement in RITeS, but they may help explain the effect of age. However, the previous analysis did not compare software and experts directly as we did, but compared both groups against a gold standard in a much smaller sample (n = 119).^30^ Given the high prevalence of these pre‐stroke features in elderly stroke populations (50–80% had at least one of these findings in RITeS) future assessments of AI software for stroke should also investigate their impact. Image quality did not affect human‐software agreement, but fewer poor‐quality scans were successfully processed and were unavailable for comparison.

### Strengths and Limitations of RITeS

According to our prespecified standard, RITeS scans represent typical populations in whom e‐ASPECTS *may* be used.^16^ Background radiological features in RITeS are comparable with published elderly population data.^40^ We controlled for between‐study differences. Using PROBAST, we found RITeS data to be low risk for bias and appropriate for validation testing of e‐ASPECTS. We had more scans successfully processed by e‐ASPECTS compared with other studies using existing data (90% vs 69%).^41^

We used expert interpretation of imaging for comparison with software which does not represent routine care and was not undertaken in real time. Interpretation of non‐enhanced CT in acute stroke is challenging, and for features such as presence of ischemia, even experts disagree, particularly when clinical information is not available as for the majority of expert imaging assessments in RITeS.^5^, ^19^ We feel it is appropriate to compare AI software against a gold‐standard due to expectations that AI performs similar to or enhances best practice. There is a risk of incorporation bias in RITeS because the index test (baseline CT) was used to derive the reference standard (final diagnosis). However, this risk is likely small because for most of our patients, follow‐up information is more likely than baseline imaging to determine the reference standard.

We reported our results using TRIPOD because e‐ASPECTS is a prediction model for diagnosis. However, TRIPOD is not ideal for RITeS. Given our focus on diagnostic accuracy testing and inclusion of meta‐analysis modeling, we have also reviewed STARD and PRISMA guidelines, respectively (Appendices S1–S3). An expert consensus statement aiming to improve legislation for radiology AI, suggests testing AI software beyond accuracy of the defined task and to test other performance (eg, reliability, when software is applied outside its designated clinical use, and how software copes with unexpected data), as we have done.^42^

On sensitivity testing with balanced representation of the 9 RITeS studies, expert versus software ASPECT scores were better matched after a large proportion of IST‐3 scans (the major contributor to RITeS) were removed. This likely reflects differences in the scan and patient parameters that were associated with lesser human‐software ASPECTS agreement and were more common in IST‐3, especially increased age, worse stroke severity, and thicker CT‐slices.

We include an up‐to‐date summary of all published evidence for e‐ASPECTS. As with most published studies (22/24), RITeS is a secondary analysis, albeit using prospectively collected data. However, we strove to minimize patient (or CT) selection in RITeS in a population designed to replicate routine care and we reported all outcomes. We did not exclude scans based on image quality. Contemporaneous CT acquisition and processing may increase the proportion successfully handled by software. In addition, common advances in CT technology that reduce scan time, improve tissue resolution, and reduce artifacts are likely to account for improvements in software processing success. However, as a surrogate for the modernity of CT hardware, we did not find that the year of CT acquisition differed between groups where software processing was or was not successful. Data shared by Brainomix from one UK hospital indicate >99% of ~ 1,800 CTs were successfully processed by e‐ASPECTS but the rates of processing routine, nonselected data are not publicly available. The design of RITeS cannot capture all potential benefits or risks of decision‐support software when used in real time. For example, whether additional information provided by software modifies clinician detection of true stroke features on CT, care pathways, or outcome. Instead, we have maximized the use of available data. Robust evidence of benefit and absence of harm are needed to confirm the enthusiasm for decision‐support AI. Our clinically relevant results should inform routine practice and guide future research.

## Conclusions

When software processing is successful, e‐ASPECTS has moderate diagnostic accuracy for stroke feature detection on CT. When used as indicated to detect acute MCA territory ischemia, e‐ASPECTS may be more sensitive but less specific than experts with more false positive results. Increased false positive results were also apparent for hemorrhage detection and among patients with a stroke mimic (even when we excluded those with visible abnormalities). We found a 10% failure rate for software processing. Our findings emphasize that e‐ASPECTS should only be used as indicated to assist *experienced* readers to identify *possible* findings and should not be used as a standalone diagnostic tool. Users should interpret software results with caution and, according to the clinical context, be capable of independently recognizing true ischemic, hemorrhagic, and mimic features on CT to counter software misclassification and if results are not provided. Users can improve software detection by inputting the side affected by stroke and by increasing image quality. Results may be less accurate in older patients and those with severe stroke. Given the rapid growth of AI software for medical imaging, it is important that early adoptions of these methods, such as for acute stroke imaging, are rigorously and independently assessed and that appropriate precedents for quality and clinical effectiveness are set. Further testing of AI software for stroke is required, especially prospective trials of clinical impact as we would expect for any new health care intervention. Ideally, these studies would include patients with suspected stroke, admitted to a range of centers reflecting different levels of expertise in stroke, randomized to clinical decisions *with* versus *without software assistance*, and should be completed before widespread software rollout.

## Authors Contributions

G.M., P.W., P.B., K.M., R.S., F.C., R.vK., M.M., N.S., and J.W. contributed to the conception and design of the study. G.M., C.M., D.D., and A.V. contributed to the acquisition and analysis of data. G.M. and J.W. drafted a significant proportion of the manuscript or figures.

## Potential Conflicts of Interest

Nothing to report.

## Statement of Independence

Following development of our research plan and sourcing funding, the RITeS Collaboration signed a software license agreement with Brainomix for use of e‐ASPECTS and bought the software using academic funds. We agreed to distinguish software testing relating to claims made by Brainomix and for software features with regulatory approval from testing outside the “indications for software use” or for features without regulatory approval. To comply with the agreement, a pre‐submission draft of this paper was shared with Brainomix, and, at their request, we minimally modified text to clarify the distinction between in and out‐of‐scope testing.

Brainomix staff and affiliates were not involved in creation of the RITeS research plan, setting aims, research conduct including image processing, statistical analysis, interpretation of data, or the writing of the paper.

## Supporting information


**Appendix S1** Supporting Information
