# Supplementary material for: External Validation of e‐ASPECTS Software for Interpreting Brain CT in Stroke
Source: Ann Neurol. 2022 Sep 23;92(6):943–57. doi: 10.1002/ana.26495 (PMC9826303; doi:10.1002/ana.26495)
Supplement: Supplementary file 1 — Appendix S1 Supporting Information [file ANA-92-943-s001.docx]

**SUPPLEMENTAL MATERIAL**

**External validation of e-ASPECTS software for interpreting brain CT in stroke**

Grant Mair^1^, Philip White^2^, Philip M Bath^3^, Keith Muir^4^, Rustam Al-Shahi Salman^1^, Chloe Martin^1^, David Dye^1^, Francesca Chappell^1^, Adam Vacek^1^, Rüdiger von Kummer^5^, Malcolm Macleod^1^, Nikola Sprigg^2^,

Joanna M Wardlaw^1,6^.

1. Centre for Clinical Brain Sciences, University of Edinburgh, UK
2. Translational and Clinical Research Institute, Newcastle University and Newcastle upon Tyne Hospitals NHS Trust, UK
3. Stroke Trials Unit, Mental Health & Clinical Neuroscience, University of Nottingham, UK
4. Institute of Neuroscience & Psychology, University of Glasgow, UK
5. Department of Neuroradiology, University Hospital, Technische Universität Dresden, Germany
6. UK Dementia Research Institute Centre at the University of Edinburgh, Edinburgh, UK

**Author for correspondence:**

Dr Grant Mair

Centre for Clinical Brain Sciences

Chancellor’s Building

University of Edinburgh

49 Little France Crescent

Edinburgh

EH16 4SB

UK

**Email:** [grant.mair@ed.ac.uk](mailto:grant.mair@ed.ac.uk)

**Supplement Table 1. Summary of published evidence for e-ASPECTS**

| Ref | Centre, Country | Design | Sample size | Aim of study | Sensitivity, specificity, Accuracy** | Software vs human agreement | Described population*** | Reported software failures | Only anterior circulation ischaemic stroke | Input lesion side | Excluded cases with artefacts/ poor quality | COI with company | Tested impact of imaging or patient factors | Other potential biases |
| --- | --- | --- | --- | --- | --- | --- | --- | --- | --- | --- | --- | --- | --- | --- |
| 1 | Kiel, Germany | R | 52 | Compare humans & other software against ground truth | e-ASPECTS: 14-34,83-99,68-72% Experts: 34-40,93-96,77% | - | Y | N | Y | NS | Y | N | N | Assessed individual ASPECTS regions as if independent |
| 2 | Rochester, USA | R | 60 | Compare humans with/out software & ground truth | Experts -, -, 78% | 0.25 weighted agreement | Incomplete: No time from onset | N | Y | NS | Y | N | N | Assessed individual ASPECTS regions as if independent |
| 3 | Brno, Czech Rep | R | 81 | Compare humans & other software against ground truth | e-ASPECTS: 41, 91, 76%  Experts: 46, 93, 79% | - | Y | N | Y | NS | Y | Y  Consultancy | N | N |
| 4 | Leuven, Belgium | R | 156 | Compare humans & ground truth, predict outcome | - | ICC 0.47 | Y | N | Y | Y | Y | N | N | N |
| 5 | Curitiba, Brazil | R | 116 | Compare humans & ground truth | e-ASPECTS 61, 96, 74%  Experts: 28-75, 69-98, 60-84% |  | Y | N | Y | NS | NS | N | N | N |
| 6 | Essen, Germany | R | 150* | Compare humans & other software |  | ICC 0.81 | Y | Y | N | Y | Y | N | N | N |
| 7 | Homburg, Germany | P | 15 | Compare humans | - | - | Y | Y | N | NS | N | Y  Authorship | N | N |
| 8 | Essen, Germany | R | 119* | Compare humans & ground truth | e-ASPECTS: 83, 57, 67%  Experts: 63-81, 76-91, 77-80% | - | Y | N | Y | NS | N | N | Y  Brain changes | Assessed individual ASPECTS regions as if independent |
| 9 | Heidelberg, Germany | R | 34* | Compare humans & ground truth | e-ASPECTS: 40, 94, -%  Experts: 16-39, 96-99, -% | - | Y | N | Y | NS | Y | Y  Consultancy, funding | N |  |
| 10 | Erlangen, Germany | R | 131 | Compare humans & other software | - | ICC 0.87 | Incomplete: No time from onset | Y | Y | Y | Y | N | N | Assessed individual ASPECTS regions as if independent |
| 11 | Ostrava, Czech Rep | P | 45 | Compare ground truth | - | - | Y | Y | Y | NS | N | N | N | Assessed individual ASPECTS regions as if independent |
| 12 | Atlanta, USA | R | 150 | As biomarker in clinical research | - | - | Incomplete: No time from stroke onset | N | N | NS | NS | N | N |  |
| 13 | Reading, UK | P | 1 | Case study of integrated COVID pathway | - | - | Y | N | Y | NS | ND | Y  Authorship | N |  |
| 14 | Heidelberg, Germany | R | 132* | Compare humans, and ground truth | e-ASPECTS: 42-44, 91-93, 85-87% Experts: 26-44, 89-97, 84-89%. | - | Incomplete: No recruitment dates | N | Y | N | Y | Y  Authorship, funding, consultancy | N |  |
| 15 | Heidelberg, Germany | R | 388* | Compare ground truth (subset) and clinical outcomes | - | - | Incomplete: No selection criteria or recruitment dates | N | Y | NS | NS | Y  Authorship, consultancy | N |  |
| 16 | Heidelberg, Germany | R | 390* | As biomarker in clinical research | - | - | Y | N | Y | NS | NS | Y  Consultancy | N |  |
| 17 | Sydney, Australia | R | 1480 | Predictor of outcome | - | - | Y | Y | Y | (Y) | Y | Y  Consultancy | N |  |
| 18 | Heidelberg, Germany | R | 258* | Impact of CT slice thickness on results | - | - | Incomplete: No time to CT | Y | Y | NS | Y | Y  Consultancy | Y  Slice thickness |  |
| 19 | Rochester, USA | R | 178 | Compare humans | - | Kappa 0.25  ICC 0.66 | Incomplete; No time to CT | N | Y | NS | Y | N | N | Assessed individual ASPECTS regions as if independent |
| 20 | Barcelona, Spain | R | 184 | Compare humans & ground truth | - | Spearman’s rank corr 0.44 | Y | N | Y | NS | Y | N | N |  |
| 21 | Heidelberg, Germany | R | 220* | Predictor of outcome | - | ICC 0.72-0.76 | Y | Y | Y | NS | Y | Y  Funding, consultancy | N |  |
| 22 | Heidelberg, Germany | R | 102* | As biomarker in clinical research | - | - | Y | N | Y | Y | Y | Y  Consultancy | Y  Imaging factors |  |
| 23 | Heidelberg, Germany | R | 43* | Assessing impact of CT reconstruction on results | - | - | Y | N | Y | NS | NS | Y  Consultancy | Y  CT recon |  |
| 24 | New York, USA | R | 58 | Compare humans & ground truth | - | Kappa 0.84 | Y | N | Y | NS | NS | Y  Advisory board for partner company | N |  |
| Total | **8 countries 15 centres** | **21 R,**  **3 P** | **4543 (3247 definitely unique)**  **Median 125 Mean 189** | **16 comparisons,**  **5 as biomarker in research,**  **2 assessed technical features,**  **1 case study** | **e-ASPECTS**  **Sens 14-83%**  **Spec 57-99%**  **Acc 67-87%**  **EXPERTS**  **Sens 16-81%**  **Spec 69-99%**  **Acc 60-89%** | **Kappa 0.25-0.84**  **ICC**  **0.47-0.87** | **17 Y,**  **7 incomplete** | **7 reported software failures,**  **17 did not** | **21 only included anterior circulation stroke,**  **3 included other patient types** | **5 input lesion side** | **14 excluded poor-quality cases,**  **3 did not,**  **7 not stated** | **14 have COI, 10 do not** | **4 tested impact of patient factors** | **8 assessed individual ASPECTS regions as if independent** |

**Notes:**

Results from PubMed search of terms: ‘e-ASPECTS’ and ‘Brainomix’, correct to 6th August 2021.

NS = not stated, R = retrospective, P = Prospective, ICC = intraclass correlation coefficient, COI = conflict of interest.

* Likely population overlap between different studies at same centres, i.e. not necessarily unique cases.

** Including at least dates for recruitment, inclusion/exclusion criteria, and clinical/demographic details of final cohort, particularly time from symptom onset.

*** Only includes estimates based on total ASPECTS (i.e. not estimates of individual ASPECTS regions). If multiple results available, only most statistically robust method presented.

**References:**

1. Austein F, Wodarg F, Jurgensen N, Huhndorf M, Meyne J, Lindner T, Jansen O, Larsen N, Riedel C. Automated versus manual imaging assessment of early ischemic changes in acute stroke: comparison of two software packages and expert consensus. Eur Radiol. 2019;29:6285-6292
2. Brinjikji W, Abbasi M, Arnold C, Benson JC, Braksick SA, Campeau N, Carr CM, Cogswell PM, Klaas JP, Liebo GB, et al. e-ASPECTS software improves interobserver agreement and accuracy of interpretation of aspects score. Interv Neuroradiol. 2021. DOI: 10.1177/15910199211011861
3. Cimflova P, Volny O, Mikulik PR, Tyshchenko B, Belaskova S, et al. Detection of ischemic changes on baseline multimodal computed tomography: expert reading vs. Brainomix and RAPID software. *J Stroke Cerebrovasc Dis*. 2020;29:104978
4. Demeestere J, Scheldeman L, Cornelissen SA, Heye S, Wouters A, et al. Alberta Stroke Program Early CT Score Versus Computed Tomographic Perfusion to Predict Functional Outcome After Successful Reperfusion in Acute Ischemic Stroke. Stroke. 2018;49:2361-2367
5. Ferreti LA, Leitao CA, Teixeira BCA, Lopes Neto FDN, VF ZE, Lange MC. The use of e-ASPECTS in acute stroke care: validation of method performance compared to the performance of specialists. Arq Neuropsiquiatr. 2020;78:757-761
6. Goebel J, Stenzel E, Guberina N, Wanke I, Koehrmann M, Kleinschnitz C, Umutlu L, Forsting M, Moenninghoff C, Radbruch A. Automated ASPECT rating: comparison between the Frontier ASPECT Score software and the Brainomix software. Neuroradiology. 2018;60:1267-1272
7. Grunwald IQ, Ragoschke-Schumm A, Kettner M, Schwindling L, Roumia S, Helwig S, et al. First Automated Stroke Imaging Evaluation via Electronic Alberta Stroke Program Early CT Score in a Mobile Stroke Unit. Cerebrovasc Dis. 2016;42:332-338
8. Guberina N, Dietrich U, Radbruch A, Goebel J, Deuschl C, Ringelstein A, Kohrmann M, Kleinschnitz C, Forsting M, Monninghoff C. Detection of early infarction signs with machine learning-based diagnosis by means of the Alberta Stroke Program Early CT score (ASPECTS) in the clinical routine. Neuroradiology. 2018;60:889-901
9. Herweh C, Ringleb PA, Rauch G, Gerry S, Behrens L, Mohlenbruch M, Gottorf R, Richter D, Schieber S, Nagel S. Performance of e-ASPECTS software in comparison to that of stroke physicians on assessing CT scans of acute ischemic stroke patients. Int J Stroke. 2016;11:438-445
10. Hoelter P, Muehlen I, Goelitz P, Beuscher V, et al. Automated ASPECT scoring in acute ischemic stroke: comparison of three software tools. Neuroradiology. 2020;62:1231-1238
11. Kral J, Cabal M, Kasickova L, Havelka J, Jonszta T, Volny O, Bar M. Machine learning volumetry of ischemic brain lesions on CT after thrombectomy-prospective diagnostic accuracy study in ischemic stroke patients. Neuroradiology. 2020;62:1239-1245
12. Landzberg D, Nogueira NG, Al-Bayati AR, Kim SJ, Bouslama M, et al. Baseline Characteristics of Patients with Symptomatic Carotid Webs: A Matched Case Control Study. J Stroke Cerebrovasc Dis. 2021;30:105823
13. Nagaratnam K, Harston G, Flossmann E, Canavan C, Geraldes RC, Edwards C. Innovative use of artificial intelligence and digital communication in acute stroke pathway in response to COVID-19. Future Healthc 2020;7:169-173; DOI: 10.7861/fhj.2020-0034
14. Nagel S, Sinha D, Day D, Reith W, Chapot R, et al. e-ASPECTS software is non-inferior to neuroradiologists in applying the ASPECT score to computed tomography scans of acute ischemic stroke patients. Int J Stroke. 2017;12:615-622
15. Nagel S, Joly O, Pfaff J, Papanagiotou P, Fassbender K, et al. e-ASPECTS derived acute ischemic volumes on non-contrast-enhanced computed tomography images. Int J Stroke. 2020;15:995-1001
16. Nagel S, Herweh C, Pfaff JAR, et al. Simplified selection criteria for patients with longer or unknown time to treatment predict good outcome after mechanical thrombectomy. Journal of NeuroInterventional Surgery 2019;11:559-562
17. Nagel S, Wang X, Carcel C, Robinson T, Lindley RI, et al. Clinical Utility of Electronic Alberta Stroke Program Early Computed Tomography Score Software in the ENCHANTED Trial Database. Stroke. 2018;49:1407-1411
18. Neuberger U, Nagel S, Pfaff J, Ringleb PA, Herweh C, et al. Impact of slice thickness on clinical utility of automated Alberta Stroke Program Early Computed Tomography Scores. Eur Radiol. 2020;30:3137-3145
19. Neuhaus A, Seyedsaadat SM, Mihal D, Benson J, Mark I, et al. Region-specific agreement in ASPECTS estimation between neuroradiologists and e-ASPECTS software. J Neurointerv Surg. 2020;12:720-723
20. Olive-Gadea M, Martins N, Boned S, Carvajal J, Moreno MJ, Muchada M, et al. Baseline ASPECTS and e-ASPECTS correlation with infarct volume and functional outcome in patients undergoing mechanical thrombectomy. Journal of Neuroimaging 2019;29:198-20
21. Pfaff J, Herweh C, Schieber S, Schonenberger S, Bosel J, et al. e-ASPECTS Correlates with and Is Predictive of Outcome after Mechanical Thrombectomy. AJNR Am J Neuroradiol. 2017;38:1594-1599
22. Purrucker JC, Mattern N, Herweh C, et al. Electronic Alberta Stroke Program Early CT score change and functional outcome in a drip-and-ship stroke service. Journal of NeuroInterventional Surgery 2020;12:252-255
23. Seker F, Pfaff J, Nagel S, et al. CT reconstruction levels affect automated and reader-based ASPECTS ratings in acute ischemic stroke. J Neuroimaging 2019;29:62- 4
24. Sundaram VK, Goldstein J, Wheelwright D, Aggarwal A, Pawha PS, et al. Automated ASPECTS in Acute Ischemic Stroke: A Comparative Analysis with CT Perfusion. AJNR Am J Neuroradiol. 2019;40:2033-2038.

**Supplement Figure 1. Forest plots for e-ASPECTS diagnostic accuracy testing.**


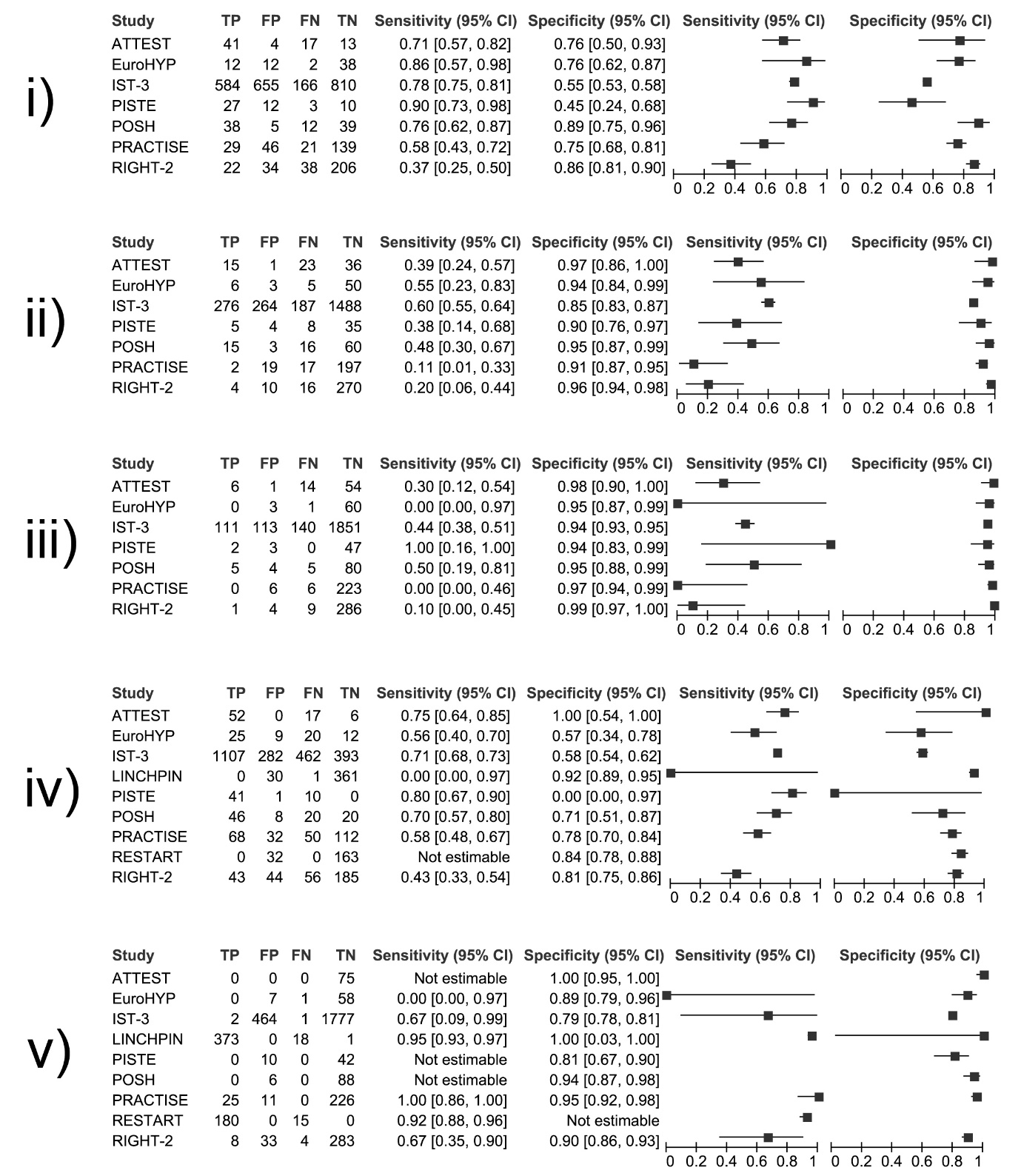


**Note:** i) e-ASPECTS 10 vs 0-9, ii) e-ASPECTS 8-10 vs 0-7, iii) e-ASPECTS 6-10 vs 0-5, iv) e-ASPECTS detecting ischaemic signs, v) e-ASPECTS detecting haemorrhage.

i)-iii) uses ‘Core’ testing dataset with 3035 cases. iv)-v) use ‘Enriched’ dataset and include cases with imaging features outside software scope: non-MCA ischaemia (116/3708, 3.1%) and structural stroke mimics (80/3708, 2.2%). TP = true positive, TN = true negative, FP = false positive, FN = false negative. See Table 2 for more details.

**Supplement Figure 2. ROC curves for e-ASPECTS diagnostic accuracy testing.**


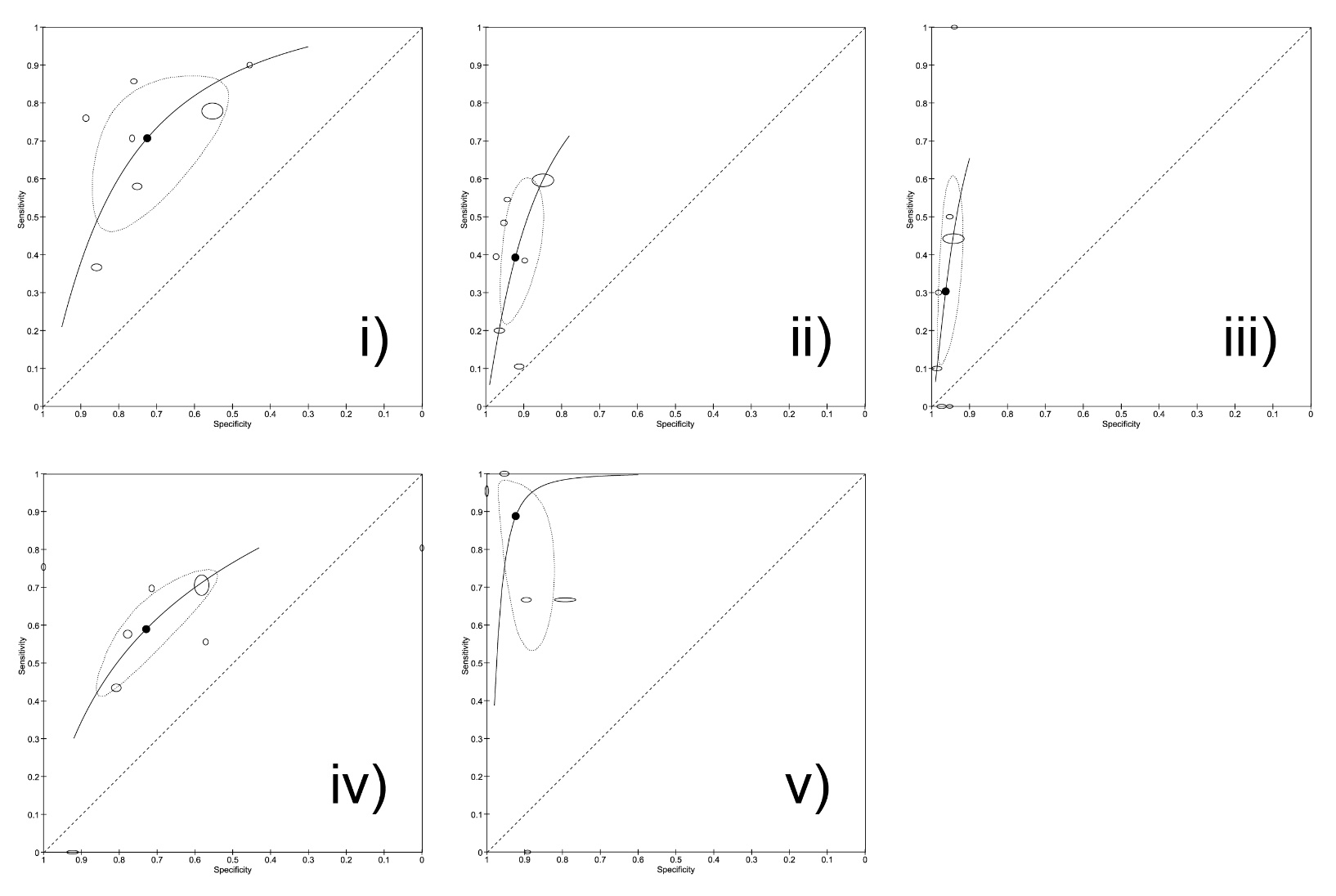


**Note:** i) e-ASPECTS 10 vs 0-9, ii) e-ASPECTS 8-10 vs 0-7, iii) e-ASPECTS 6-10 vs 0-5, iv) e-ASPECTS detecting ischaemic signs, v) e-ASPECTS detecting haemorrhage. iv)-v) use ‘Enriched’ dataset and include cases with imaging features outside software scope: non-MCA ischaemia (116/3708, 3.1%) and structural stroke mimics (80/3708, 2.2%). See Table 2 for more details.

Open circles are individual study results proportional to sample size, closed circles are summary results. Dotted lines enclose 95% confidence regions.

**Appendix 1. TRIPOD (Transparent reporting of a multivariable prediction model for individual prognosis or diagnosis) Checklist: Prediction model validation.**

| **Section/ Topic** | **Item Item** | **Checklist Item** | | **Page** |
| --- | --- | --- | --- | --- |
| **Title and abstract** | | | | |
| Title | 1 | Identify the study as developing and/or validating a multivariable prediction model, the target population, and the outcome to be predicted. | | Cover |
| Abstract | 2 | Provide a summary of objectives, study design, setting, participants, sample size, predictors, outcome, statistical analysis, results, and conclusions. | | 2 |
| **Introduction** | | | | |
| Background and objectives | 3a | | Explain the medical context (including whether diagnostic or prognostic) and rationale for developing or validating the multivariable prediction model, including references to existing models. | 3 |
|  | 3b | | Specify the objectives, including whether the study describes the development or validation of the model or both. | 3 |
| **Methods** | | | | |
| Source of data | 4a | | Describe the study design or source of data (e.g., randomized trial, cohort, or registry data), separately for the development and validation data sets, if applicable. | 4 |
|  | 4b | | Specify the key study dates, including start of accrual; end of accrual; and, if applicable, end of follow-up. | 4 |
| Participants | 5a | | Specify key elements of the study setting (e.g., primary care, secondary care, general population) including number and location of centres. | 4 |
|  | 5b | | Describe eligibility criteria for participants. | 4-5 |
|  | 5c | | Give details of treatments received, if relevant. | 4 |
| Outcome | 6a | | Clearly define the outcome that is predicted by the prediction model, including how and when assessed. | 3 |
|  | 6b | | Report any actions to blind assessment of the outcome to be predicted. | 5 |
| Predictors | 7a | | Clearly define all predictors used in developing or validating the multivariable prediction model, including how and when they were measured. | 5 |
|  | 7b | | Report any actions to blind assessment of predictors for the outcome and other predictors. | 5 |
| Sample size | 8 | | Explain how the study size was arrived at. | 4 |
| Missing data | 9 | | Describe how missing data were handled (e.g., complete-case analysis, single imputation, multiple imputation) with details of any imputation method. | 6 |
| Statistical analysis methods | 10c | | For validation, describe how the predictions were calculated. | 4 |
|  | 10d | | Specify all measures used to assess model performance and, if relevant, to compare multiple models. | 4-6 |
|  | 10e | | Describe any model updating (e.g., recalibration) arising from the validation, if done. | - |
| Risk groups | 11 | | Provide details on how risk groups were created, if done. | - |
| Development vs. validation | 12 | | For validation, identify any differences from the development data in setting, eligibility criteria, outcome, and predictors. | - |
| **Results** | | | | |
| Participants | 13a | | Describe the flow of participants through the study, including the number of participants with and without the outcome and, if applicable, a summary of the follow-up time. A diagram may be helpful. | Fig 1 |
|  | 13b | | Describe the characteristics of the participants (basic demographics, clinical features, available predictors), including the number of participants with missing data for predictors and outcome. | 7 |
|  | 13c | | For validation, show a comparison with the development data of the distribution of important variables (demographics, predictors and outcome). | - |
| Model performance | 16 | | Report performance measures (with CIs) for the prediction model. | 7-8 |
| Model-updating | 17 | | If done, report the results from any model updating (i.e., model specification, model performance). | - |
| **Discussion** | | | | |
| Limitations | 18 | | Discuss any limitations of the study (such as nonrepresentative sample, few events per predictor, missing data). | 10-11 |
| Interpretation | 19a | | For validation, discuss the results with reference to performance in the development data, and any other validation data. | 9-10 |
|  | 19b | | Give an overall interpretation of the results, considering objectives, limitations, results from similar studies, and other relevant evidence. | 9,11 |
| Implications | 20 | | Discuss the potential clinical use of the model and implications for future research. | 9-11 |
| **Other information** | | | | |
| Supplementary information | 21 | | Provide information about the availability of supplementary resources, such as study protocol, Web calculator, and data sets. | Refs, 12 |
| Funding | 22 | | Give the source of funding and the role of the funders for the present study. | 6, 12 |

**Appendix 2. STARD (STAndards for Reporting Diagnostic accuracy studies) Guideline.**

| **Section & Topic** | **No** | **Item** | **Reported on page #** |
| --- | --- | --- | --- |
| **TITLE OR ABSTRACT** |  |  |  |
|  | **1** | Identification as a study of diagnostic accuracy using at least one measure of accuracy  (such as sensitivity, specificity, predictive values, or AUC) | 1 |
| **ABSTRACT** |  |  |  |
|  | **2** | Structured summary of study design, methods, results, and conclusions  (for specific guidance, see STARD for Abstracts) | 1 |
| **INTRODUCTION** |  |  |  |
|  | **3** | Scientific and clinical background, including the intended use and clinical role of the index test | 3 |
|  | **4** | Study objectives and hypotheses | 3 |
| **METHODS** |  |  |  |
| ***Study design*** | **5** | Whether data collection was planned before the index test and reference standard  were performed (prospective study) or after (retrospective study) | 4-5 |
| ***Participants*** | **6** | Eligibility criteria | 4-5 |
|  | **7** | On what basis potentially eligible participants were identified  (such as symptoms, results from previous tests, inclusion in registry) | 4-5 |
|  | **8** | Where and when potentially eligible participants were identified (setting, location and dates) | 4 |
|  | **9** | Whether participants formed a consecutive, random or convenience series | 4 |
| ***Test methods*** | **10a** | Index test, in sufficient detail to allow replication | 5 |
|  | **10b** | Reference standard, in sufficient detail to allow replication | 4-6 |
|  | **11** | Rationale for choosing the reference standard (if alternatives exist) | 4, 10 |
|  | **12a** | Definition of and rationale for test positivity cut-offs or result categories  of the index test, distinguishing pre-specified from exploratory | 5-6, 9 |
|  | **12b** | Definition of and rationale for test positivity cut-offs or result categories  of the reference standard, distinguishing pre-specified from exploratory | 6, 9 |
|  | **13a** | Whether clinical information and reference standard results were available  to the performers/readers of the index test | - |
|  | **13b** | Whether clinical information and index test results were available  to the assessors of the reference standard | 5 |
| ***Analysis*** | **14** | Methods for estimating or comparing measures of diagnostic accuracy | 6 |
|  | **15** | How indeterminate index test or reference standard results were handled | 4-5 |
|  | **16** | How missing data on the index test and reference standard were handled | 6 |
|  | **17** | Any analyses of variability in diagnostic accuracy, distinguishing pre-specified from exploratory | 6 |
|  | **18** | Intended sample size and how it was determined | 4-5 |
| **RESULTS** |  |  |  |
| ***Participants*** | **19** | Flow of participants, using a diagram | Fig 1 |
|  | **20** | Baseline demographic and clinical characteristics of participants | 7, Table 1, Supp Table 3 |
|  | **21a** | Distribution of severity of disease in those with the target condition | 7, Table 1 |
|  | **21b** | Distribution of alternative diagnoses in those without the target condition | 7, Supp Table 3 |
|  | **22** | Time interval and any clinical interventions between index test and reference standard | 7 |
| ***Test results*** | **23** | Cross tabulation of the index test results (or their distribution)  by the results of the reference standard | - |
|  | **24** | Estimates of diagnostic accuracy and their precision (such as 95% confidence intervals) | 8, Table 2 |
|  | **25** | Any adverse events from performing the index test or the reference standard | - |
| **DISCUSSION** |  |  |  |
|  | **26** | Study limitations, including sources of potential bias, statistical uncertainty, and generalisability | 10-11 |
|  | **27** | Implications for practice, including the intended use and clinical role of the index test | 2, 9-11 |
| **OTHER INFORMATION** |  |  |  |
|  | **28** | Registration number and name of registry | - |
|  | **29** | Where the full study protocol can be accessed | 6 |
|  | **30** | Sources of funding and other support; role of funders | 1, 6, 12 |

From: Bossuyt PM, Reitsma JB, Bruns DE, Gatsonis CA, et al. STARD Group. STARD 2015: An updated list of essential items for reporting diagnostic accuracy studies. BMJ 2015;351:h5527. Doi: 10.1136/bmj.h5527.

**Appendix 3. PRISMA (Preferred Reporting Items for Systematic Reviews and Meta-Analyses) 2020 Checklist.**

| **TITLE** | | | **Page** |
| --- | --- | --- | --- |
| Title | 1 | Identify the report as a systematic review. | - |
| **ABSTRACT** | | |  |
| Abstract | 2 | See the PRISMA 2020 for Abstracts checklist. | 1 |
| **INTRODUCTION** | | |  |
| Rationale | 3 | Describe the rationale for the review in the context of existing knowledge. | 2-3 |
| Objectives | 4 | Provide an explicit statement of the objective(s) or question(s) the review addresses. | 6 |
| **METHODS** | | |  |
| Eligibility criteria | 5 | Specify the inclusion and exclusion criteria for the review and how studies were grouped for the syntheses. | 4-5 |
| Information sources | 6 | Specify all databases, registers, websites, organisations, reference lists and other sources searched or consulted to identify studies. Specify the date when each source was last searched or consulted. | - |
| Search strategy | 7 | Present the full search strategies for all databases, registers and websites, including any filters and limits used. | - |
| Selection process | 8 | Specify the methods used to decide whether a study met the inclusion criteria of the review, including how many reviewers screened each record and each report retrieved, whether they worked independently, and if applicable, details of automation tools used in the process. | - |
| Data collection process | 9 | Specify the methods used to collect data from reports, including how many reviewers collected data from each report, whether they worked independently, any processes for obtaining or confirming data from study investigators, and if applicable, details of automation tools used in the process. | - |
| Data items | 10a | List and define all outcomes for which data were sought. Specify whether all results that were compatible with each outcome domain in each study were sought (e.g. for all measures, time points, analyses), and if not, the methods used to decide which results to collect. | 5-6 |
|  | 10b | List and define all other variables for which data were sought (e.g. participant and intervention characteristics, funding sources). Describe any assumptions made about any missing or unclear information. | - |
| Study risk of bias assessment | 11 | Specify the methods used to assess risk of bias in the included studies, including details of the tool(s) used, how many reviewers assessed each study and whether they worked independently, and if applicable, details of automation tools used in the process. | 6 |
| Effect measures | 12 | Specify for each outcome the effect measure(s) (e.g. risk ratio, mean difference) used in the synthesis or presentation of results. | 6 |
| Synthesis methods | 13a | Describe the processes used to decide which studies were eligible for each synthesis (e.g. tabulating the study intervention characteristics and comparing against the planned groups for each synthesis (item #5)). | - |
|  | 13b | Describe any methods required to prepare the data for presentation or synthesis, such as handling of missing summary statistics, or data conversions. | - |
|  | 13c | Describe any methods used to tabulate or visually display results of individual studies and syntheses. | 6 |
|  | 13d | Describe any methods used to synthesize results and provide a rationale for the choice(s). If meta-analysis was performed, describe the model(s), method(s) to identify the presence and extent of statistical heterogeneity, and software package(s) used. | 6 |
|  | 13e | Describe any methods used to explore possible causes of heterogeneity among study results (e.g. subgroup analysis, meta-regression). | 6 |
|  | 13f | Describe any sensitivity analyses conducted to assess robustness of the synthesized results. | 6 |
| Reporting bias assessment | 14 | Describe any methods used to assess risk of bias due to missing results in a synthesis (arising from reporting biases). | - |
| Certainty assessment | 15 | Describe any methods used to assess certainty (or confidence) in the body of evidence for an outcome. | - |
| **RESULTS** | | |  |
| Study selection | 16a | Describe the results of the search and selection process, from the number of records identified in the search to the number of studies included in the review, ideally using a flow diagram. | - |
|  | 16b | Cite studies that might appear to meet the inclusion criteria, but which were excluded, and explain why they were excluded. | - |
| Study characteristics | 17 | Cite each included study and present its characteristics. | 4-5 |
| Risk of bias in studies | 18 | Present assessments of risk of bias for each included study. | App 4 |
| Results of individual studies | 19 | For all outcomes, present, for each study: (a) summary statistics for each group (where appropriate) and (b) an effect estimate and its precision (e.g. confidence/credible interval), ideally using structured tables or plots. | Table 2, Supp Figs 1-2 |
| Results of syntheses | 20a | For each synthesis, briefly summarise the characteristics and risk of bias among contributing studies. | 8 |
|  | 20b | Present results of all statistical syntheses conducted. If meta-analysis was done, present for each the summary estimate and its precision (e.g. confidence/credible interval) and measures of statistical heterogeneity. If comparing groups, describe the direction of the effect. | 8, Table 2, Supp Figs 1-2 |
|  | 20c | Present results of all investigations of possible causes of heterogeneity among study results. | - |
|  | 20d | Present results of all sensitivity analyses conducted to assess the robustness of the synthesized results. | 7-8 |
| Reporting biases | 21 | Present assessments of risk of bias due to missing results (arising from reporting biases) for each synthesis assessed. | - |
| Certainty of evidence | 22 | Present assessments of certainty (or confidence) in the body of evidence for each outcome assessed. | 8 |
| **DISCUSSION** | | |  |
| Discussion | 23a | Provide a general interpretation of the results in the context of other evidence. | 9-11 |
|  | 23b | Discuss any limitations of the evidence included in the review. | 10-11 |
|  | 23c | Discuss any limitations of the review processes used. | - |
|  | 23d | Discuss implications of the results for practice, policy, and future research. | 2, 9-11 |
| **OTHER INFORMATION** | | |  |
| Registration and protocol | 24a | Provide registration information for the review, including register name and registration number, or state that the review was not registered. | - |
|  | 24b | Indicate where the review protocol can be accessed, or state that a protocol was not prepared. | 6 |
|  | 24c | Describe and explain any amendments to information provided at registration or in the protocol. | - |
| Support | 25 | Describe sources of financial or non-financial support for the review, and the role of the funders or sponsors in the review. | 1, 6, 12 |
| Competing interests | 26 | Declare any competing interests of review authors. | 12 |
| Availability of data, code and other materials | 27 | Report which of the following are publicly available and where they can be found: template data collection forms; data extracted from included studies; data used for all analyses; analytic code; any other materials used in the review. | 12 |

From: Page MJ, McKenzie JE, Bossuyt PM, Boutron I, Hoffmann TC, Mulrow CD, et al. The PRISMA 2020 statement: an updated guideline for reporting systematic reviews. BMJ 2021;372:n71. doi: 10.1136/bmj.n71

**Appendix 4. PROBAST (Prediction model study Risk Of Bias Assessment Tool).**

**Step 1: Specify your systematic review question**

| State your systematic review question to facilitate the assessment of the applicability of the evaluated models to your question. *The following table should be completed once per systematic review.* |
| --- |

| **Criteria** | **Specify your systematic review question** |
| --- | --- |
| *Intended use of model:* | Validation of artificial intelligence software (e-ASPECTS by Brainomix Ltd.) designed to automate the identification of imaging features indicative of stroke on CT brain scans (and thus assist human readers to correctly diagnose stroke) |
| ***Participants*** *including selection criteria and setting:* | Patients presenting acutely to hospital with symptoms and signs of stroke, and where baseline CT brain imaging is acquired. |
| ***Predictors*** *(used in prediction modelling), including types of predictors (e.g. history, clinical examination, biochemical markers, imaging tests), time of measurement, specific measurement issues (e.g., any requirements/ prohibitions for specialized equipment):* | Three CT imaging biomarkers of stroke assessed by e-ASPECTS are 1) ischaemic brain lesions, 2) arterial blood clots (dense arteries), and 3) brain haemorrhage. |
| *Outcome to be predicted:* | Final diagnosis of ischaemic or haemorrhagic stroke |

**Step 2: Classify the type of prediction model evaluation**

| Use the following table to classify the evaluation as model development, model validation or model update, or combination. Different signalling questions apply for different types of prediction model evaluation. If the evaluation does not fit one of these classifications then PROBAST should not be used. |
| --- |

| **Classify the evaluation based on its aim** | | | |
| --- | --- | --- | --- |
| **Type of prediction study** | **PROBAST boxes to complete** | **Tick as appropriate** | **Definition for type of prediction model study** |
| Development only | Development |  | Prediction model development without external validation. These studies may include internal validation methods, such as bootstrapping and cross-validation techniques. |
| Development and validation | Development and validation |  | Prediction model development combined with external validation in other participants in the same article. |
| Validation only | Validation | 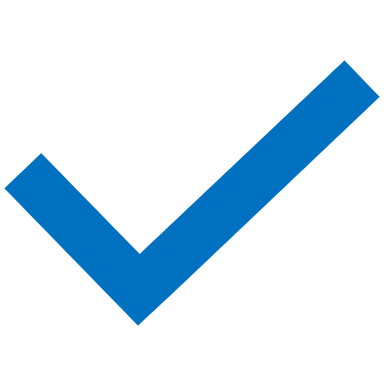 | External validation of existing (previously developed) model in other participants. |

| *This table should be completed once for each publication being assessed and for each relevant outcome in your review.*  **1. ATTEST** | |  |
| --- | --- | --- |
| **Publication reference** | Huang X, Cheripelli BK, Lloyd SM, Kalladka D, Moreton FC, Siddiqui A, Ford I, Muir KW. Alteplase versus tenecteplase for thrombolysis after ischaemic stroke (ATTEST): a phase 2, randomised, open-label, blinded endpoint study. *Lancet Neurol*. 2015;14:368-376 | |
| **Models of interest** | e-ASPECTS | |
| **Outcome of interest** | Final diagnosis of ischaemic or haemorrhagic stroke, or stroke mimic | |

| **2. EuroHYP** | |  |
| --- | --- | --- |
| **Publication reference** | van der Worp HB, Macleod MR, Bath PM, Demotes J, Durand-Zaleski I, Gebhardt B, Gluud C, Kollmar R, Krieger DW, Lees KR, et al. EuroHYP-1: European multicenter, randomized, phase III clinical trial of therapeutic hypothermia plus best medical treatment vs. best medical treatment alone for acute ischemic stroke. *Int J Stroke*. 2014;9:642-645 | |
| **Models of interest** | e-ASPECTS | |
| **Outcome of interest** | Final diagnosis of ischaemic or haemorrhagic stroke, or stroke mimic | |

| **3. IST-3** | |  |
| --- | --- | --- |
| **Publication reference** | IST-3 Collaborative Group. The benefits and harms of intravenous thrombolysis with recombinant tissue plasminogen activator within 6 h of acute ischaemic stroke (the third international stroke trial [IST-3]): a randomised controlled trial. *Lancet*. 2012;379:2352-2363 | |
| **Models of interest** | e-ASPECTS | |
| **Outcome of interest** | Final diagnosis of ischaemic or haemorrhagic stroke, or stroke mimic | |

| **4. LINCHPIN** | |  |
| --- | --- | --- |
| **Publication reference** | Samarasekera N, Lerpiniere C, Fonville AF, Farrall AJ, Wardlaw JM, White PM, Torgersen A, Ironside JW, Smith C, Al-Shahi Salman R, et al. Consent for Brain Tissue Donation after Intracerebral Haemorrhage: A Community-Based Study. *PLoS One*. 2015;10:e0135043 | |
| **Models of interest** | e-ASPECTS | |
| **Outcome of interest** | Final diagnosis of ischaemic or haemorrhagic stroke, or stroke mimic | |

| **5. PISTE** | |  |
| --- | --- | --- |
| **Publication reference** | Muir KW, Ford GA, Messow CM, Ford I, Murray A, Clifton A, Brown MM, Madigan J, Lenthall R, Robertson F, et al. Endovascular therapy for acute ischaemic stroke: the Pragmatic Ischaemic Stroke Thrombectomy Evaluation (PISTE) randomised, controlled trial. *J Neurol Neurosurg Psychiatry*. 2017;88:38-44 | |
| **Models of interest** | e-ASPECTS | |
| **Outcome of interest** | Final diagnosis of ischaemic or haemorrhagic stroke, or stroke mimic | |

| **6. POSH** | |  |
| --- | --- | --- |
| **Publication reference** | MacDougall NJ, McVerry F, Huang X, Welch A, Fulton R, Muir K. Post-stroke hyperglycaemia is associated with adverse evolution of acute ischaemic injury. *Cerebrovasc Dis*. 2014;37(suppl 1):267 | |
| **Models of interest** | e-ASPECTS | |
| **Outcome of interest** | Final diagnosis of ischaemic or haemorrhagic stroke, or stroke mimic | |

| **7. PRACTISE** | |  |
| --- | --- | --- |
| **Publication reference** | El-Tawil S, Wardlaw J, Ford I, Mair G, Robinson T, Kalra L, Muir KW. Penumbra and re-canalization acute computed tomography in ischemic stroke evaluation: PRACTISE study protocol. *Int J Stroke*. 2017;12:671-678 | |
| **Models of interest** | e-ASPECTS | |
| **Outcome of interest** | Final diagnosis of ischaemic or haemorrhagic stroke, or stroke mimic | |

| **8. RESTART** | |  |
| --- | --- | --- |
| **Publication reference** | RESTART Collaboration. Effects of antiplatelet therapy after stroke due to intracerebral haemorrhage (RESTART): a randomised, open-label trial. *Lancet*. 2019;393:2613-2623 | |
| **Models of interest** | e-ASPECTS | |
| **Outcome of interest** | Final diagnosis of ischaemic or haemorrhagic stroke, or stroke mimic | |

| **9. RIGHT-2** | |  |
| --- | --- | --- |
| **Publication reference** | RIGHT-2 Investigators. Prehospital transdermal glyceryl trinitrate in patients with ultra-acute presumed stroke (RIGHT-2): an ambulance-based, randomised, sham-controlled, blinded, phase 3 trial. *Lancet*. 2019;393:1009-1020 | |
| **Models of interest** | e-ASPECTS | |
| **Outcome of interest** | Final diagnosis of ischaemic or haemorrhagic stroke, or stroke mimic | |

**Step 3: Assess risk of bias and applicability**

| PROBAST is structured as four key domains. Each domain is judged for risk of bias (low, high or unclear) and includes signalling questions to help make judgements. Signalling questions are rated as yes (Y), probably yes (PY), probably no (PN), no (N) or no information (NI). All signalling questions are phrased so that “yes” indicates absence of bias. Any signalling question rated as “no” or “probably no” flags the potential for bias; you will need to use your judgement to determine whether the domain should be rated as “high”, “low” or “unclear” risk of bias. The guidance document contains further instructions and examples on rating signalling questions and risk of bias for each domain.  The first three domains are also rated for concerns regarding applicability (low/ high/ unclear) to your review question defined above.  *Complete all domains separately for each evaluation of a distinct model. Shaded boxes indicate where signalling questions do not apply and should not be answered.* |
| --- |

| **DOMAIN 1: Participants** | | | |
| --- | --- | --- | --- |
| **A. Risk of Bias** | | | |
| *Describe the sources of data and criteria for participant selection:*  All studies with imaging available to RITeS aimed to identify patients presenting acutely to hospital with symptoms of stroke. Most studies recruited patients with ischaemic stroke (ATTEST, EuroHYP, IST-3, PISTE, POSH, PRACTISE). RESTART and LINCHPIN recruited patients with haemorrhagic stroke. With ambulance-based recruitment (i.e. pre-hospital recruitment before brain imaging), RIGHT-2 ultimately included a mix of ischaemic stroke, haemorrhagic stroke, and stroke mimics. Patient selection for ATTEST, EuroHYP, IST-3, PISTE, POSH, PRACTISE, RESTART, and RIGHT-2 was based on individual trial inclusion/exclusion criteria. LINCHPIN included all consecutive patients in the local geographical region diagnosed with intracerebral haemorrhage over 2 years, with no exclusion criteria.  Case selection for RITeS differed between the available studies. For 6/9 of the RITeS studies (ATTEST, EuroHYP, IST-3, PISTE, POSH, PRACTISE) all cases with an available baseline CT and/or CTA scan were included. For the remaining 3/9 RITeS studies (LINCHPIN, RESTART, RIGHT-2) we used subsamples. For LINCHPIN and RESTART, cases were sequentially selected (ordered by trial ID) until sufficient numbers of haemorrhagic stroke were included. For RIGHT-2 we included all cases with a final diagnosis of mimic in addition to a random subsample of 150 cases with a final diagnosis of ischaemic stroke.  Case selection was not related to imaging features or results for any of the RITeS studies. | | | |
|  | | Dev | Val |
| - 1. Were appropriate data sources used, e.g. cohort, RCT or nested case-control study data? | |  | Y |
| - 1. Were all inclusions and exclusions of participants appropriate? | |  | Y |
| **Risk of bias introduced by selection of participants** | **RISK:**  *(low/ high/ unclear)* |  | **LOW** |
| *Rationale of bias rating:* | | | |
| Despite differing requirements of individual RITeS studies (which may help to improve the range of available patient characteristics), all recruited patients were clinically diagnosed with ischaemic, haemorrhagic or mimic stroke and were therefore relevant for RITeS.  Imaging was not used to select cases for RITeS.  Ultimately, only some patients had visible imaging biomarkers of stroke and this proportion was not controlled and thus expected to replicate routine practice. | | | |
| **B. Applicability** | | | |
| *Describe included participants, setting and dates:*  All patients recruited to RITeS collaborative studies presented acutely to hospital with symptoms of stroke and had baseline CT imaging for assessment.  e-ASPECTS software was developed using the CT scans of patients presenting acutely to hospital with symptoms of stroke. Although the specific cases used for software development are not publically available, Brainomix is a UK-based company, a spin out from Oxford University. It is highly likely that the imaging of local (UK) stroke patients were used in development. All RITeS studies included UK recruitment, but several were international thus increasing the diversity and wider applicability of patients used for assessment. | | | |
| **Concern that the included participants and setting do not match the review question** | **CONCERN:**  *(low/ high/ unclear)* |  | **LOW** |
| *Rationale of applicability rating:* | | | |
| High likelihood that development (Brainomix) and validation (RITeS) patients have similar demographics.  All patients are the same clinically. | | | |

| **DOMAIN 2: Predictors** | | | |
| --- | --- | --- | --- |
| **A. Risk of Bias** | | | |
| *List and describe predictors included in the final model, e.g. definition and timing of assessment:*  Three e-ASPECTS predictors are considered:   1. Ischaemic brain lesions (within middle cerebral artery, MCA territory, with lesion extent conveyed using ASPECT scoring where 10 = no MCA lesion, and 0 = entire MCA affected) 2. Dense arteries (a surrogate measure of arterial occlusion, e-ASPECTS assesses MCA branches and the internal carotid artery, ICA which supplies MCA) 3. Brain haemorrhage (presence vs absence, if present defined as a volume).   All 3 of these same predictors were also scored by our (reference standard) expert human readers. Predictors 1 and 2 were scored identically, 3 was scored by humans as presence/absence but volume was only estimated (and is therefore not included in our analysis).  In all cases, predictor assessment was based only on baseline CT imaging. This means CT imaging acquired at initial presentation to hospital assessed while masked to all other data. | | | |
|  | | Dev | Val |
| - 1. Were predictors defined and assessed in a similar way for all participants? | |  | Y |
| - 1. Were predictor assessments made without knowledge of outcome data? | |  | Y |
| - 1. Are all predictors available at the time the model is intended to be used? | |  | Y |
| **Risk of bias introduced by predictors or their assessment** | **RISK:**  *(low/ high/ unclear)* |  | **LOW** |
| *Rationale of bias rating:*  All 3 predictors are available on baseline CT, human and software scoring was identical and was conducted blind to outcome. | | | |
| **B. Applicability** | | | |
| Concern that the definition, assessment or timing of predictors in the model do not match the review question | **CONCERN:**  *(low/ high/ unclear)* |  | **LOW** |
| *Rationale of applicability rating:*  Definition, assessment and timing of predictors are an excellent match with the review question. | | | |

| **DOMAIN 3: Outcome** | | | |
| --- | --- | --- | --- |
| **A. Risk of Bias** | | | |
| *Describe the outcome, how it was defined and determined, and the time interval between predictor assessment and outcome determination:*  Outcome was final diagnosis for patients, either ischaemic stroke or haemorrhagic stroke. Final diagnosis was determined by clinical experts at follow-up using all available information including the results of any clinical tests used such as additional brain imaging (when ischaemic stroke lesions are much clearer). Owing to the retrospective use of RITeS data for this assessment, outcome determination occurred before predictor assessment. | | | |
|  | | Dev | Val |
| - 1. Was the outcome determined appropriately? | |  | Y |
| - 1. Was a pre-specified or standard outcome definition used? | |  | Y |
| - 1. Were predictors excluded from the outcome definition? | |  | Y |
| - 1. Was the outcome defined and determined in a similar way for all participants? | |  | Y |
| - 1. Was the outcome determined without knowledge of predictor information? | |  | Y |
| - 1. Was the time interval between predictor assessment and outcome determination appropriate? | |  | Y |
| **Risk of bias introduced by the outcome or its determination** | **RISK:**  *(low/ high/ unclear)* |  | **LOW** |
| *Rationale of bias rating:*  Outcome assessment was consistent, is reflective of standard practice, and was conducted completely separate from e-ASPECTS predictor acquisition. | | | |
| **B. Applicability** | | | |
| *At what time point was the outcome determined:*  In routine clinical practice, final diagnosis would usually be defined during the first 1-2 weeks after presentation to hospital. This is also true in RITeS but was additionally confirmed at dedicated 3- or 6-month follow-up.  *If a composite outcome was used, describe the relative frequency/distribution of each contributing outcome:* | | | |
| **Concern that the outcome, its definition, timing or determination do not match the review question** | **CONCERN:**  *(low/ high/ unclear)* |  | **LOW** |
| *Rationale of applicability rating:*  Outcome assessment is based on routine clinical practice.  Good match for review question. | | | |

| **DOMAIN 4: Analysis** | | | |
| --- | --- | --- | --- |
| **Risk of Bias** | | | |
| *Describe numbers of participants, number of candidate predictors, outcome events and events per candidate predictor:*  4100 unique participants, each with a baseline CT scan. Total RITeS sample identified to be representative of routine clinical practice.  All 3 predictors possible in all participant scans but present in fewer: 34% (1390) had MCA ischaemia, 19% (768) had dense artery, 16% (643) had haemorrhage. These figure representative of routine practice.  Outcome available for all participants. | | | |
| *Describe how the model was developed (for example in regards to modelling technique (e.g. survival or logistic modelling), predictor selection, and risk group definition):*  Not applicable, our assessment validation not development. | | | |
| *Describe whether and how the model was validated, either internally (e.g. bootstrapping, cross validation, random split sample) or externally (e.g. temporal validation, geographical validation, different setting, different type of participants):*  Externally validated in a different, large group of participants. RITeS participants were separately assessed for UK clinical representation but include participants from several international studies, thus also enabling geographical validation. | | | |
| *Describe the performance measures of the model, e.g. (re)calibration, discrimination, (re)classification, net benefit, and whether they were adjusted for optimism:*  Not applicable, our assessment validation not development. | | | |
| *Describe any participants who were excluded from the analysis:*  No participants from the RITeS representative sample were excluded from the analysis. | | | |
| *Describe missing data on predictors and outcomes as well as methods used for missing data:*  Approximately 10% (429/4100) of participant CT scans failed software processing, therefore no predictors available for these participants. We reported but did not impute missing data.  Outcome data (final diagnosis) was complete for all participants. | | | |
|  | | Dev | Val |
| - 1. Were there a reasonable number of participants with the outcome? | |  | Y |
| - 1. Were continuous and categorical predictors handled appropriately? | |  | Y |
| - 1. Were all enrolled participants included in the analysis? | |  | Y |
| - 1. Were participants with missing data handled appropriately? | |  | Y |
| - 1. Was selection of predictors based on univariable analysis avoided? | |  |  |
| - 1. Were complexities in the data (e.g. censoring, competing risks, sampling of controls) accounted for appropriately? | |  | Y |
| - 1. Were relevant model performance measures evaluated appropriately? | |  | Y |
| - 1. Were model overfitting and optimism in model performance accounted for? | |  |  |
| - 1. Do predictors and their assigned weights in the final model correspond to the results from multivariable analysis? | |  |  |
| **Risk of bias introduced by the analysis** | **RISK:**  *(low/ high/ unclear)* |  | **LOW** |
| *Rationale of bias rating:*  Large number of participants, with clinically appropriate representation of predictors. Outcome available for all participants. No excluded participants, missing data reported. | | | |

**Step 4: Overall assessment**

| Use the following tables to reach overall judgements about risk of bias and concerns regarding applicability of the prediction model evaluation (development and/or validation) across all assessed domains.  *Complete for each evaluation of a distinct model.*   \| **Reaching an overall judgement about risk of bias of the prediction model evaluation** \| \| \| --- \| --- \| \| **Low risk of bias** \| If all domains were rated low risk of bias.  If a prediction model was developed without any external validation, and it was rated as low risk of bias for all domains, consider downgrading to **high risk of bias**. Such a model can only be considered as low risk of bias, if the development was based on a very large data set and included some form of internal validation. \| \| **High risk of bias** \| If at least one domain is judged to be at **high risk of bias**. \| \| **Unclear risk of bias** \| If an unclear risk of bias was noted in at least one domain and it was low risk for all other domains. \|  \| **Reaching an overall judgement about applicability of the prediction model evaluation** \| \| \| --- \| --- \| \| **Low concerns regarding applicability** \| If low concerns regarding applicability for all domains, the prediction model evaluation is judged to have **low concerns regarding applicability**. \| \| **High concerns regarding applicability** \| If high concerns regarding applicability for at least one domain, the prediction model evaluation is judged to have **high concerns regarding applicability**. \| \| **Unclear concerns regarding applicability** \| If unclear concerns (but no “high concern”) regarding applicability for at least one domain, the prediction model evaluation is judged to have **unclear concerns regarding applicability** overall. \| |
| --- | --- | --- | --- | --- | --- | --- | --- | --- | --- | --- | --- | --- | --- | --- | --- | --- |

| **Overall judgement about risk of bias and applicability of the prediction model evaluation** | | |
| --- | --- | --- |
| **Overall judgement of risk of bias** | **RISK:**  *(low/ high/ unclear)* | **LOW** |
| *Summary of sources of potential bias:* | | |
| **Overall judgement of applicability** | **CONCERN:**  *(low/ high/ unclear)* | **LOW** |
| *Summary of applicability concerns:* | | |
